# Mitochondrial Dysfunction in Neurodegenerative Diseases: Molecular Mechanisms, Primary Drivers Versus Early Amplifiers, and Emerging Mitochondria-Targeted Therapeutic Strategies

**DOI:** 10.3390/ijms27219993

**Published:** 2026-11-07

**Authors:** Prarthana Kalerammana Gopalakrishna, Thirupathirao Vishnumukkala, Zaw Myo Hein, Barani Karikalan, Srikumar Chakravarthi, Farida Hussan, Riya Sandeep Deoghare, Saravanan Jagadeesan, Narendiran Krishnasamy, Soe Ei Phyu, Pamela David, Mohd Amir Kamaruzzaman

**Affiliations:** 1 Physiology Discipline, Human Biology Division, School of Medicine, IMU University, 126, Jln Jalil Perkasa 19, Bukit Jalil, Kuala Lumpur 57000, Federal Territory of Kuala Lumpur, Malaysia; prarthana@imu.edu.my; 2 Anatomy Discipline, Human Biology Division, School of Medicine, IMU University, 126, Jln Jalil Perkasa 19, Bukit Jalil, Kuala Lumpur 57000, Federal Territory of Kuala Lumpur, Malaysia; thirupathirao@imu.edu.my; 3 Department of Basic Medical Sciences, College of Medicine, Ajman University, Ajman P.O. Box 346, United Arab Emirates; z.hein@ajman.ac.ae; 4 Department of Pathology, Faculty of Medicine, MAHSA University, Bandar Saujana Putra, Jenjarom 42610, Selangor, Malaysia; barani@mahsa.edu.my; 5 Faculty of Medicine, Nursing and Health Sciences, SEGi University, 9, Jalan Teknologi, Pju 5 Kota Damansara, Petaling Jaya 47810, Selangor, Malaysia; srikumarc@segi.edu.my; 6 Department of Basic Medical Sciences, Faculty of Medicine and Health Sciences, Universiti Malaysia Sarawak, Jln Datuk Mohammad Musa, Kota Samarahan 94300, Sarawak, Malaysia; khinpapah@gmail.com; 7 School of Medicine, IMU University, 126, Jln Jalil Perkasa 19, Bukit Jalil, Kuala Lumpur 57000, Federal Territory of Kuala Lumpur, Malaysia; 00000044360@student.imu.edu.my; 8 Department of Anatomy, School of Medicine, Taylor’s University, Lakeside Campus, 1, Jln Taylors, Subang Jaya 47500, Selangor, Malaysia; saravanan.jagadeesan@taylors.edu.my; 9 Clinical Skills Suite, School of Medicine, Taylor’s University, Lakeside Campus, 1, Jln Taylors, Subang Jaya 47500, Selangor, Malaysia; narendiran.krishnasamy@taylors.edu.my; 10 Department of Basic Medical Sciences, Faculty of Medicine, University of Cyberjaya, Persiaran Bestari, Cyber 11, Cyberjaya 63000, Selangor, Malaysia; soe@cyberjaya.edu.my; 11 Department of Anatomy, Faculty of Medicine, Universiti Malaya, Kuala Lumpur 57000, Federal Territory of Kuala Lumpur, Malaysia; rosiepamela@um.edu.my; 12 Department of Anatomy, Faculty of Medicine, Universiti Kebangsaan Malaysia (UKM), Jalan Yaacob Latif, Bandar Tun Razak, Cheras, Kuala Lumpur 57000, Federal Territory of Kuala Lumpur, Malaysia

**Keywords:** mitochondrial dysfunction, mitophagy, mitochondrial dynamics, oxidative phosphorylation, mitochondria-targeted antioxidants, neurodegeneration

## Abstract

Mitochondria are critical for bioenergetics, calcium and redox homeostasis, and apoptosis in neurons. Neurons are especially susceptible to mitochondrial malfunction since they are terminally differentiated and have extraordinary energy requirements. Mechanisms such as defective oxidative phosphorylation, altered fission-fusion dynamics, defective mitophagy, impaired axonal transport and excessive reactive oxygen species (ROS) generation are increasingly appreciated as important mechanisms in major neurodegenerative diseases (NDDs) like Alzheimer’s disease (AD), Parkinson’s disease (PD), amyotrophic lateral sclerosis (ALS) and Huntington’s disease (HD). The review discusses the structure and function of mitochondria with emphasis on oxidative phosphorylation, ROS and reactive nitrogen species (RNS) signalling, calcium-mediated excitotoxicity, lipid metabolism, and apoptotic and non-apoptotic cell-death mechanisms including necroptosis, ferroptosis, pyroptosis and parthanatos. In particular, disease-specific processes are highlighted, such as impaired mitophagy by amyloid β/tau in AD, PTEN-induced kinase 1 (PINK1)/Parkin dysfunction and complex I inhibition in PD, Superoxide Dismutase 1 (SOD1)/C9orf72-associated mitochondrial defects in ALS, and bioenergetic dysfunction by mutant huntingtin in HD. Therapeutic approaches include mitochondria-targeted antioxidants, fusion and biogenesis enhancers, fission inhibitors, inflammasome modulators, Mitochondrial Permeability Transition Pore (mPTP) inhibitors and gene therapy. Also highlighted are recent advancements such as Ubiquitin-Specific Peptidase 30 (USP30) inhibition, intercellular mitochondrial transfer and regulatory approval of a mitochondria-targeted peptide, together with evidence supporting causality and hurdles underpinning clinical translation. Graded against human-genetic, patient-tissue and model-based evidence, mitochondrial dysfunction is best supported as a primary driver in PD and genetic ALS, and as an early amplifier of proteinopathic or mutant-huntingtin toxicity in sporadic AD and HD.

## 1. Introduction

Neurons are the primary signalling entities of the nervous system, tasked with receiving, processing, and transferring information throughout intricate neural networks. Their anatomical and functional specializations, such as elongated axons and complex dendritic arborizations, facilitate swift communication and signal integration over considerable distances within the body. Mature neurons, in contrast to numerous other cell types, are terminally differentiated, highly polarized, and predominantly irreplaceable, highlighting the imperative to preserve their functional integrity throughout life [1].

A characteristic attribute of neurons is their exceptional energy requirement, chiefly to sustain ion gradients vital for synaptic transmission, axonal transport, and membrane potential. About 20% of the body’s overall oxygen and glucose utilization is designated for the brain, with neurons constituting the predominant portion of this metabolic function [2]. Mitochondria, the cellular organelles commonly known as the “powerhouses of the cell”, are essential for fulfilling these elevated bioenergetic demands via oxidative phosphorylation. Mitochondria, besides ATP synthesis, govern calcium homeostasis, reactive oxygen species (ROS) equilibrium, apoptosis, and cellular signalling, which are essential processes for maintaining neuronal viability and plasticity [3].

Mitochondrial dysfunction is a recurrent feature of neurodegenerative disorders, such as Alzheimer’s disease (AD), Parkinson’s disease (PD), amyotrophic lateral sclerosis (ALS), and Huntington’s disease (HD). These illnesses are marked by gradual and selective neuronal degeneration, frequently resulting in cognitive and motor deficits that profoundly affect quality of life and impose a considerable socioeconomic burden worldwide [4]. Mitochondrial dysfunction in neurodegeneration is characterized by various pathogenic processes, including impaired oxidative phosphorylation, increased ROS generation, altered mitochondrial dynamics (fission and fusion), disordered mitophagy, and decreased mitochondrial axonal transport. These disruptions hinder synaptic function, elevate neuronal vulnerability to excitotoxicity, and activate apoptotic pathways, which, depending on the disease and its stage, may initiate, amplify or follow neuronal injury (Section 10.1) [5,6]. The aim of this review is to compare shared and disease-selective mitochondrial defects in AD, PD, ALS and HD, to grade the evidence that they are causal, and to explain why mitochondria-targeted therapies have not yet succeeded clinically.

In AD, mitochondrial dysfunction is detectable before clinical symptoms and accompanies amyloid-β and tau pathology [7]; the supporting evidence is largely observational or model-based (tiers ii and iii, Section 10.1) rather than genetic. Mutations in the nuclear genes PINK1, PRKN, and PARK7, which encode proteins involved in mitochondrial quality control, impair mitochondrial quality control in PD resulting in the degeneration of dopaminergic neurons in the substantia nigra [8]. Similarly, ALS and HD are characterized by mitochondrial transport abnormalities and bioenergetic dysfunction in motor neurons and striatal neurons, respectively [9]. In HD, mutant huntingtin is the primary insult, and mitochondrial impairment appears to amplify rather than initiate striatal degeneration (Section 8). Unlike predominantly disease-specific reviews, this article integrates shared and disease-selective mitochondrial defects across AD, PD, ALS, and HD and relates these mechanisms to the translational maturity of emerging therapies. Particular emphasis is placed on blood–brain barrier penetration, target engagement, disease-stage heterogeneity, and biomarker-guided patient stratification.

Mitochondrial dysfunction in neurodegeneration has been reviewed extensively, including broad overviews of mitochondrial biology in health and disease [3,10], syntheses focused on individual disorders such as AD [6] and PD [11], and analyses of single therapeutic classes such as mitochondria-targeted antioxidants [12] or mitochondrial medicine [13]. These works generally address a single disease, mechanism or drug class. They rarely ask how strong the evidence is that mitochondrial defects are causal rather than secondary. Nor do they ask why interventions that work in experimental models have failed in patients.

The present review therefore differs from earlier work in four respects. First, it compares AD, PD, ALS and HD within a single mechanistic framework, distinguishing defects that are shared across diseases from those that are disease selective. Second, it grades the evidence for a causal role of mitochondria in each disease according to whether the defect is supported by human genetics, by patient tissue or biomarker data that precede neuronal loss, or only by experimental models (Section 10.1). Third, it examines why mitochondria-targeted therapies that are effective in preclinical systems have not yet produced disease-modifying benefit in patients, including recently recognised pitfalls such as off-target activity of the widely used DRP1 inhibitor mdivi-1 [14] and the PINK1-independence of much basal mitophagy in vivo [15,16], and it compares the main mitochondria-targeted antioxidants. Fourth, it integrates developments including the entry of a USP30 inhibitor into clinical testing, microglia-to-neuron mitochondrial transfer and the first regulatory approval of a mitochondria-targeted peptide.

## 2. Methodology

This review was conducted using a comprehensive narrative literature review methodology to assess the role of mitochondrial dysfunction in the development of major neurodegenerative diseases and to describe potential mitochondria-targeted treatment options. This narrative review searched PubMed/MEDLINE, Scopus, Web of Science, and Google Scholar from inception to 31 March 2026. English-language peer-reviewed articles addressing mitochondrial dysfunction in major neurodegenerative diseases and mitochondria-targeted therapeutic strategies were considered. Updated searches were completed in October 2026 to find any new publications since the last search was performed. These included searches for articles mentioned during peer review. This review did not follow a systematic protocol and was not registered; no PRISMA flow diagram was produced and the number of records screened at title and abstract level was not tallied. Records were selected by reading titles, abstracts, and where relevant, full texts for relevance to the thematic areas listed below.

The search method integrated Medical Subject Headings (MeSH) terms and free-text keywords pertinent to mitochondrial dysfunction and neurodegenerative disorders. The primary search terms encompassed: “mitochondria”, “mitochondrial dysfunction”, “oxidative phosphorylation”, “reactive oxygen species”, “mitophagy”, “mitochondrial dynamics”, “mitochondrial fission”, “mitochondrial fusion”, “calcium homeostasis”, “apoptosis”, “Alzheimer’s disease”, “Parkinson’s disease”, “amyotrophic lateral sclerosis”, “Huntington’s disease”, “neurodegeneration”, “mitochondria-targeted therapy”, “MitoQ”, “SS-31”, “Mdivi-1”, and “gene therapy”. Boolean operators (“AND”, “OR”) were employed to enhance search combinations and optimize the retrieval of pertinent studies.

Original research articles, systematic reviews, meta-analyses, clinical trials, translational studies, and experimental investigations utilizing cellular, animal, and human models were deemed eligible for inclusion. Studies were chosen for their pertinence to mitochondrial architecture and function, mitochondrial quality control processes, disease-specific mitochondrial pathology, and treatment strategies aimed at mitochondrial pathways. Articles deficient in scientific quality, conference abstracts missing full-text access, publications in languages other than English, and studies irrelevant to neurodegenerative illnesses were removed. Priority was given to peer-reviewed human genetic, post-mortem and clinical studies, followed by animal and cellular models. Sources were appraised narratively for study design, relevance of the model to human disease, replication by independent groups and consistency across studies, and no formal risk-of-bias tool was applied. Sponsor announcements and regulatory press releases were used only to document trial registration or regulatory status and not as evidence of efficacy.

The collected evidence was thematically synthesized and categorized into principal areas, encompassing mitochondrial bioenergetics, oxidative stress, calcium dysregulation, apoptotic and non-apoptotic cell death pathways, mitochondrial dynamics, mitophagy, mitochondrial biogenesis, and disease-specific mechanisms in AD, PD, ALS, and HD. Novel treatment strategies addressing mitochondrial dysfunction were also critically discussed. This study aimed to deliver a comprehensive and current overview of mitochondrial pathophysiology in neurodegenerative illnesses and to pinpoint potential directions for future translational research and therapeutic advancement. The strength of the evidence for each disease was graded with the three-tier scheme described in Section 10.1.

## 3. Mitochondria: Structure and Function

According to current theories, mitochondria evolved from free living bacteria and participated in the origin of eukaryotic cells via a process termed endosymbiosis [17]. This concept posits that initial anaerobic eukaryotic cells assimilated primitive mitochondria, fostering a mutually beneficial association for both organisms. As a consequence of this symbiotic association, mitochondria greatly enhanced cellular energy production by generating adenosine triphosphate (ATP) through the respirator chain, while the host cell, in turn, offered a stable environment conducive to bacterial proliferation [18].

The discovery of mitochondrial DNA (mtDNA) in the 1960s added support to this theory. MtDNA is a double-stranded, circular molecule with a unique genetic code. It exists in several copies within cells, corresponding to the energy requirements of the tissue. The genetic code is not the same as nuclear DNA; for example, tryptophan and methionine are encoded by distinct codons. This indicates that many evolutionary alterations failed to preserve mtDNA within the organelle [19]. Over time, most mitochondrial genes were either lost or moved to nuclear DNA. Consequently, human mtDNA contains 37 genes: 13 protein-coding genes, 22 tRNA genes, and 2 rRNA genes [20]. However, the preservation of a separate mitochondrial genome as a remnant of this evolutionary origin also makes mitochondria particularly vulnerable to genetic mutations, which can lead to significant pathogenic consequences. Mutations in mitochondrial DNA can cause significant pathogenic effects due to differences in inheritance between mitochondrial and nuclear DNA, leading to homoplasmy and heteroplasmy, both of which influence disease phenotypes. Homoplasmy is the condition where mtDNA in a cell or organism is identical, either normal or mutated, often resulting in severe clinical manifestations. In contrast, heteroplasmy describes the coexistence of normal and mutated mtDNA, with the proportion of mutant genomes determining the severity and variability of mitochondrial diseases [19,21].

Reflecting this evolutionary origin, mitochondria are double-membraned organelles, often referred to as the powerhouses of eukaryotic cells, that play a crucial role in cellular energy production and other essential metabolic processes. These organelles perform various functions to regulate cellular homeostasis [22]. Understanding the structural organization of mitochondria is fundamental to appreciating their physiological significance in cellular homeostasis and organismal health. They are dynamic entities capable of altering their shape, size, and quantity through processes such as fission and fusion, enabling them to adjust to cellular energy requirements. Mitochondria have two different membranes: the outer mitochondrial membrane (OMM) and the inner mitochondrial membrane (IMM). They also have two different compartments: the intermembrane space (IMS) and the matrix. Every component of the mitochondrion is specialised in executing distinct functions, establishing a highly efficient system for energy production and metabolic regulation [10].

The OMM is a smooth, lipid-enriched surface consisting of ancestral beta barrel proteins that serve as the interface between the mitochondrion and cytosol. It is relatively permeable due to the presence of porins, voltage-dependent anion-selective channels (VDACs), which allow the passage of small molecules, ions and metabolites up to 10 kDa in size. The outer mitochondrial membrane serves as a protective barrier and enables the exchange of materials between the mitochondrion and the cellular environment [23]. It also includes enzymes implicated in many metabolic pathways, including fatty acid elongation and phospholipid synthesis. The OMM contains alpha-helical membrane proteins like MFF (mitochondrial fission factor) and mitochondrial Rho GTPase (MIRO), which play important roles in regulating mitochondrial dynamics and cellular communication. Through their involvement in the mitochondrial division dynamin-related protein machinery and mitochondrial motility, the OMM serves as a key regulator of mitochondrial behaviour. In addition, the OMM is essential for mitochondrial dynamics, encompassing fission and fusion, and acts as a signalling hub for multiple signalling pathways, especially those related to apoptosis [24,25].

IMM is highly impermeable and extends into the interior (or matrix) of the organelle, forming extensive invaginations known as cristae. These cristae significantly increase the surface area and house the machinery of electron transport chain (ETC) and ATP synthase, key players in oxidative phosphorylation. The potential rationale for the folding of the IMM is to augment the surface area available for energy production. Furthermore, the IMM is dense in cardiolipin, a unique phospholipid that strengthens membrane integrity and facilitates the function of respiratory chain complexes [26,27].

The intermembrane space is situated between the OMM and the IMM and contains proteins that participate in signalling and metabolic processes. This compartment is crucial for maintaining the proton gradient created during the electron transport chain, as protons are transported from the matrix to the intermembrane space by respiratory complexes. In addition, it contains several protein components vital for mitochondrial and cellular metabolism, including the regulation of programmed cell death and energy conversion. Mitochondrial Intermembrane Space Assembly (MIA) machinery is a specialized system that mediates the import, oxidative folding, and consists of the assembly of a subset of IMS proteins such as cysteine-rich proteins like TIM proteins [28]. The MIA system is characterized by a regulated thiol-disulfide exchange mechanism that facilitates correct protein folding and stability via disulfide bond formation, distinguishing it from other mitochondrial protein import processes [29].

The mitochondrial matrix, surrounded by the inner mitochondrial membrane (IMM), is a gel-like compartment that houses enzymes essential for the primary processes of oxidative metabolism. It serves as the site of the Krebs cycle/tricarboxylic acid (TCA) cycle, producing high-energy electron carriers that fuel the electron transport chain (ETC). It sustains a negative membrane potential, establishing an electrochemical gradient, thereby facilitating ATP production. In addition, it harbours enzymes that participate in additional metabolic processes, including β-oxidation of fatty acids and the components of the urea cycle [30].

### 3.1. Process of ATP Production Through Oxidative Phosphorylation

Mitochondria generate the majority of cellular ATP through oxidative phosphorylation (OXPHOS), a process that occurs at the IMM and represents the final stage of cellular respiration, following glycolysis and the TCA cycle. It harnesses energy stored in reduced electron carriers, such as NADH and FADH2, which are produced during the TCA cycle and other metabolic pathways. These carriers donate electrons to ETC; a series of protein complexes embedded in the IMM [31].

The ETC consists of four multi-subunit complexes (I–IV) and two mobile electron carriers such as coenzyme Q (ubiquinone) and cytochrome c, which sequentially transfer electrons from NADH and FADH2 to molecular oxygen, the final electron acceptor. Electron transfer through the ETC is an exergonic process that drives the active transport of protons from the mitochondrial matrix into the intermembrane space, generating an electrochemical proton gradient across the IMM. This gradient, known as the proton motive force, provides the energy required for ATP synthesis [32].

ATP synthase, also known as Complex V, harnesses the proton motive force to catalyse the phosphorylation of ADP to ATP. The tight coupling between electron transport and ATP synthase ensures efficient energy transfer, with oxidative phosphorylation optimally yielding approximately 30–32 ATP per glucose molecule under optimal conditions, surpassing the ATP yield from glycolysis alone. The rate of OXPHOS is closely controlled by cellular energy demand, and depends on the availability of ADP, inorganic phosphate, oxygen, and reducing substrates like NADH. Uncoupling proteins can dissipate the proton gradient as heat, reducing ATP production for thermogenesis. ROS, by-products of the ETC, can also modulate mitochondrial function and signaling, though excessive ROS production may lead to oxidative stress [32].

Mitochondrial OXPHOS also contributes to cellular homeostasis beyond ATP generation. Excess ROS from electron leakage is discussed in Section 3.2 [33]. Tissues like the brain require substantial energy; therefore, efficient mitochondrial ATP production is very important for normal cell and organ function. Dysfunction of oxidative phosphorylation resulting from mtDNA mutations, compromised electron transport chain (ETC) components, or oxidative damage is linked to energy failure and plays a role in the pathogenesis of metabolic and neurodegenerative disorders, such as PD, AD, HD, and ALS [34].

The production of ATP is the most important role of mitochondria; nevertheless, these organelles also perform a wide variety of other functions that are essential for cellular homeostasis. Through their involvement in cellular metabolism, calcium homeostasis, regulation of ROS, and programmed cell death, mitochondria play a critical role in maintaining the survival and function of cells. Neuronal mitochondria must therefore meet an exceptionally high energy demand [35].

### 3.2. Reactive Oxygen Species

ROS are by-products of enzymatic reactions across various cellular compartments and play dual roles in maintaining cellular homeostasis and contributing to cellular dysfunction and disease pathophysiology. Enzymes like nicotinamide adenine dinucleotide phosphate [NADPH] oxidases (NOXs) produce ROS for signalling purposes, where ROS potentially participate in normal physiological processes or contribute to maladaptive responses depending on their cellular source and tissue environment [36,37].

Mitochondria are crucial regulators of aerobic energy production, which require a balance between pro-oxidant and antioxidant systems. Mitochondrial ROS (mitoROS) are a natural by-product of mitochondrial respiration; however, dysregulated mitoROS production is closely associated with inflammatory and metabolic diseases. Within the electron transport chain, complexes I to III serve as entry points for electrons derived from NADH [38]. At complex I, superoxide (O_2_•^−^) is produced through the interaction of molecular O_2_ with reduced flavin mononucleotide (FMN), while complex III produces low levels of O_2_•^−^ under physiological conditions. In the presence of Antimycin A, the Qi (quinone reduction) site is inhibited, promoting O_2_•^−^production from the Qo (quinol oxidation) site. Superoxide generated at complex III is primarily expelled into the intermembrane space, whereas hydrogen peroxide (H_2_O_2_), produced via superoxide dismutation, is capable of diffusing into the mitochondrial matrix and adjacent compartments [39].

Complex II connects the tricarboxylic acid (TCA) cycle to the electron transport chain by turning succinate into fumarate and ubiquinone into a less reactive form. It can also help make mitoROS, especially when electron flow through complexes I and III is slow [40]. In addition to ETC complexes, many mitochondrial dehydrogenases that play a role in intermediary metabolism are also sources of mitoROS. The α-ketoglutarate dehydrogenase (KGDHC) and pyruvate dehydrogenase (PDC) complexes produce ROS via both forward electron transfer and reverse electron transport (RET). Post-translational modifications, like protein S-glutathionylation, can alter the activity and capacity of these complexes to generate ROS. Depending on the redox state of the cell, these modifications can either lower or raise ROS production [41].

#### Reactive Nitrogen Species

Nitric oxide (NO) is synthesised from L-arginine by 3 isoforms of nitric oxide synthase (NOS). NO can react with superoxide to form peroxynitrite (ONOO^−^) at a rate close to diffusion controlled. Peroxynitrite is the major reactive nitrogen species associated with nitrosative stress causing mitochondrial injury [42]. NO reversibly inhibits cytochrome c oxidase by competing with O_2_•^−^ and reducing respiration. Nitric oxide donor treatment has been shown to depolarise mitochondria in cultured hippocampal neurons via a NOS dependent mechanism [43]. Peroxynitrite has been implicated in irreversibly inhibiting the respiratory chain complexes as well as other mitochondrial proteins via tyrosine nitration and the oxidation of thiols [42].

NO has been implicated in regulating mitochondrial fission and mitophagy. Nitric oxide mediated mitochondrial fission in neurons requires dynamin-related GTPases [44]. S-nitrosylation of DRP1 enhances its GTPase activity, leading to increased fission in cells treated with Aβ and in AD brain tissue [45]. S-nitrosylation of Parkin impairs its E3 ligase function and has been observed in PD brain [46]. S-nitrosylation of PINK1 decreases PINK1/Parkin mediated mitophagy in human iPSC derived PD models [47,48]. Therefore nitrosative modifications to these proteins could impair mitochondrial fission and mitophagy. Different NOS isoforms may be involved in causing mitochondrial dysfunction. Neurons express nNOS, which associates with NMDA receptors via PSD-95, allowing it to be regulated by calcium influx secondary to glutamate stimulation [49]. This is likely the isoform responsible for mitochondrial depolarisation following excitotoxicity (see Section 3.3). Glial cells and other cells of the innate immune system express iNOS in response to inflammation and will produce higher amounts of NO over a longer period of time. It has been shown that NOS/NO can regulate innate immune cells including neutrophils [50]; this may play a role in the neuroinflammation seen in AD and PD (Section 9.4 and Section 10.3). Endothelial cells express eNOS and produce lower levels of NO, which can increase mitochondrial biogenesis via cGMP and PGC-1α [51]. Nitric oxide concentrations can also affect how cells undergo death (Section 3.4.3) [52].

### 3.3. Role of Calcium

Calcium (Ca^2+^) is necessary for maintaining cellular balance and is important for metabolism, intracellular signalling, and cell survival. Although the endoplasmic reticulum (ER) serves as the primary intracellular calcium reservoir, mitochondria are equally important regulators of calcium handling. The mitochondrial electron transport chain (ETC) creates a proton gradient that moves calcium into the mitochondrial matrix through the mitochondrial calcium uniporter (MCU). This high-capacity uniporter allows calcium to flow rapidly, while slower mechanisms maintain mitochondrial calcium homeostasis. However, excessive mitochondrial calcium accumulation results in mitochondrial calcium overload, which opens the mitochondrial permeability transition pore (mPTP), causes loss of small solutes (<1.5 kDa), causes damage to mitochondria, and eventually kills the cell. These pathways are significantly associated with ischaemic injury in the heart and brain, as well as with neurodegenerative disorders [53,54].

The identification of the mitochondrial calcium uniporter (MCU) in 2011 marked a significant advancement in the comprehension of mitochondrial calcium signalling following decades of research. MCU is a highly conserved 40-kDa protein found in the inner mitochondrial membrane and in many other organs. Functional investigations show that MCU is necessary for mitochondria to take in calcium [55,56]. Downregulation of MCU significantly diminishes mitochondrial calcium influx subsequent to ER calcium release, whereas MCU overexpression augments calcium uptake, underscoring its essential function in calcium homeostasis. In neurons, stringent regulation of mitochondrial calcium is crucial due to elevated energy requirements and dependence on calcium-mediated synaptic signalling. MCU dysfunction or excessive calcium influx causes the dysregulation of mitochondrial calcium, which leads to oxidative stress, apoptosis, and mitochondrial dysfunction in neurodegenerative diseases like PD, AD, and ALS [57].

Glutamate receptor families play a crucial role in neuronal calcium control, especially N-methyl-D-aspartate (NMDA) receptors and Ca^2+^-permeable AMPA receptors, which both allow for significant calcium influx during synaptic transmission. These receptors are crucial for synaptic regulation; nevertheless, extended glutamate exposure leads to Ca^2+^-dependent cell death, a phenomenon termed excitotoxicity [58,59]. Importantly, calcium toxicity depends on its route of cellular entry. According to Michael Tymianski’s concept of source specificity, calcium influx via L-type voltage-gated Ca^2+^ channels is comparatively non-toxic, but equivalent calcium entry via NMDA receptors results in marked neurotoxicity [60,61]. This phenomenon arises from the close association between NMDA receptors and neuronal nitric oxide synthase (nNOS) through the scaffolding protein PSD-95 [49]. Localised calcium microdomains activate nNOS, producing nitric oxide (NO), which, when combined with increased intracellular Ca^2+^ concentrations, becomes extremely lethal. Disruption of nNOS signalling or its interaction with NMDA receptors might thereby alleviate Ca^2+^-dependent neuronal damage [61].

Further refinement of this concept shows that excessive activation of extrasynaptic glutamate receptors generates toxic calcium loads leading to mitochondrial Ca^2+^-dependent damage, whereas synaptic receptor activation produces localized calcium elevations that promote neuronal survival. Pathological conditions such as glutamate spillover, decreased astrocytic uptake, or reversal of glutamate transporters can overstimulate extrasynaptic receptors, leading to neuronal damage [62,63]. A defining feature of Ca^2+^-mediated toxicity is delayed deregulation of calcium homeostasis (DCD), characterized by a secondary rise in intracellular Ca^2+^ occurring 1–10 min after glutamate exposure. This event coincides with the collapse of mitochondrial membrane potential (Δψm), linking plasma membrane signalling directly to mitochondrial dysfunction. When hippocampal neurons were grown in culture, NOS inhibition prevented glutamate from decreasing Δψm but did not change the amount of Ca^2+^ in the cytosol. A NO donor had no effect on its own but could cause large mitochondrial depolarisation when cytosolic Ca^2+^ was elevated, therefore Ca^2+^ and NO work together to decrease Δψm [64]. Large mitochondrial depolarisation was also associated with an inability to reestablish Ca^2+^ homeostasis after the removal of glutamate in these neuron cultures [65]. Since these studies used non-selective NOS inhibitors, it is believed that nNOS is involved due to its association with NMDA receptors [49,66].

Loss of mitochondrial membrane potential represents a critical irreversible step toward neuronal death, as neurons possess limited capacity to compensate for impaired oxidative phosphorylation through glycolysis. Mitochondrial depolarisation occurs mainly due to calcium excess that surpasses the buffering capacity of the mitochondrial matrix and overwhelms sodium–calcium exchange systems. Direct measurement of mitochondrial free Ca^2+^ during this process remains technically challenging due to the limitations of genetically encoded and small-molecule calcium indicators, which are affected by cytosolic calcium surges and intracellular pH alterations [67]. Interestingly, transient exposure to mitochondrial uncouplers when glutamate is stimulating the brain can preserve neurons by inhibiting mitochondrial calcium uptake at critical junctures. Even though cytosolic Ca^2+^ levels may transiently increase, preventing mitochondrial calcium accumulation promotes neuronal survival, which shows that mitochondrial Ca^2+^ uptake is a major cause of excitotoxic cell death [68,69].

### 3.4. Apoptosis

Apoptosis, the best-characterised form of programmed cell death (PCD), is essential for the development of multicellular organisms, and when dysregulated, can contribute to degenerative disease. Mitochondria are responsive to numerous stress signals. Severe ATP depletion disrupts ion gradients and Ca^2+^ handling and can promote opening of the mitochondrial permeability transition pore, so that energy failure can act as a trigger of regulated cell death [53,70]. Mitochondria make ROS, which can damage components such as the membranes, DNA, and proteins. Other stress signals that cause cell death include the misfolding of proteins, and signals from other cells, such as toxins, can send signals to the mitochondria, which can then trigger cell death [70].

A network of proteins and signalling molecules, such as cytochrome c, Bcl-2 family proteins, SMAC/Diablo, and AIF, controls the mitochondrial pathway of apoptosis. Cytochrome c is a protein found in mitochondria that is released into the cytosol when the outer membrane of the mitochondria becomes permeable (MOMP). The Bcl-2 family controls apoptosis by controlling mitochondrial outer MOMP. Pro-apoptotic proteins, like Bax and Bak, promote MOMP, while anti-apoptotic proteins, like Bcl-2 and Bcl-xL, inhibit it by trapping and neutralizing pro-apoptotic members. The balance between pro- and anti-apoptotic members determines whether MOMP occurs and the cell dies or survives [71,72].

Apoptosis proceeds through extrinsic (death-receptor) and intrinsic (mitochondrial) pathways that converge on caspase activation, and BCL-2 family proteins control the mitochondrial pathway through MOMP [71,73,74].

#### 3.4.1. Extrinsic Pathway

**Figure 1:**
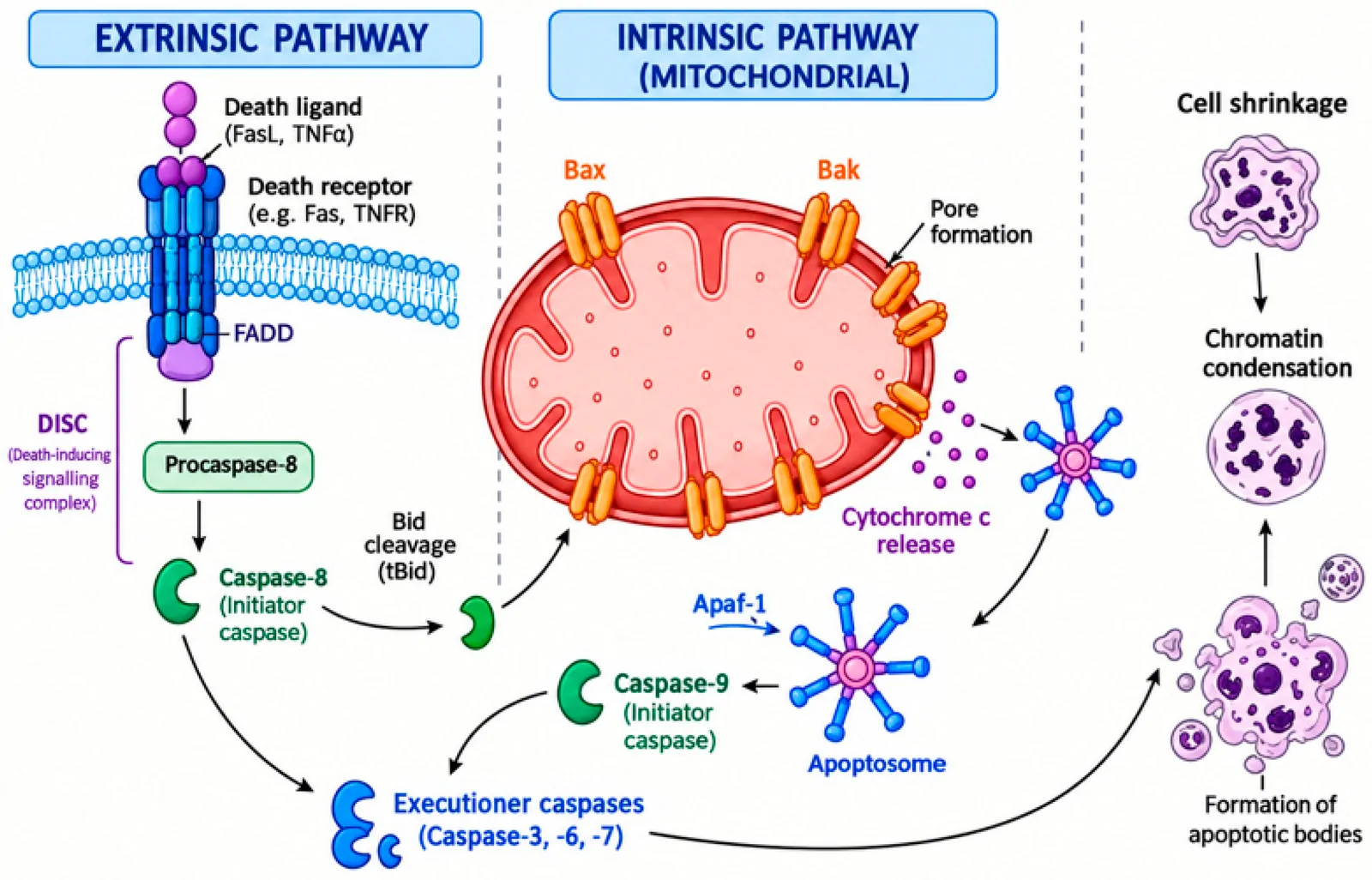
Extrinsic and intrinsic apoptotic pathways converging on mitochondria. The extrinsic (death receptor) pathway is initiated by the binding of a death ligand to its cognate death receptor (e.g., Fas, TNFR) at the plasma membrane, leading to assembly of the death-inducing signalling complex (DISC), activation of procaspase-8, and the generation of active caspase-8. Caspase-8 cleaves BID to truncated BID (tBID), which translocates to the mitochondrial outer membrane to promote BAX/BAK oligomerisation and pore formation. The intrinsic (mitochondrial) pathway is activated directly by intracellular stress signals, also triggering BAX/BAK-mediated pore formation. Both pathways converge on mitochondrial outer membrane permeabilisation (MOMP), releasing cytochrome into the cytosol. Cytochrome c associates with APAF-1 and procaspase-9 to form the apoptosome, activating executioner caspases-3, -6, and -7. Downstream morphological consequences include cell shrinkage, chromatin condensation, and the fragmentation of the dying cell into membrane-enclosed apoptotic bodies. Arrows denote directional signalling flow.

Death ligands found outside of the cell can trigger the extrinsic pathway of apoptosis. TNF and the Fas ligand are examples of these ligands. These ligands bind to the death receptors, which are members of the TNF receptor superfamily [74]. Adaptor molecules are then recruited upon receptor binding, such as Fas-Associated via Death Domain (FADD) and Tumor Necrosis Factor Receptor-1-Associated Death Domain (TRADD). These will go on to activate caspase-8, an initiator caspase. Caspase-3 is activated by caspase-8. FLICE-inhibitory protein (FLIP) inhibits caspase-8 at the death-inducing signaling complex (DISC) [75] (Figure 1).

#### 3.4.2. Intrinsic Pathway

The intrinsic pathway of apoptosis, also known as the mitochondrial pathway, is triggered when the intracellular environment is altered by either endogenous or exogenous stressors. The exogenous stressors include toxins and radiation, and endogenous stressors like DNA damage and redox imbalances, mitochondrial translocation, p53 activation, and the overexpression of BH3-only proteins [76]. Release of intermembrane-space proteins after loss of outer-membrane integrity is the pivotal step and is tightly controlled by the BCL-2 family. Activation of pro-apoptotic proteins BAX and BAK leads to oligomerization, mitochondrial outer membrane permeabilization (MOMP) [71]. In addition, cytochrome c is released into the cytosol and binds with apoptotic protease-activating factor 1 (Apaf-1) to form the apoptosome complex, which activates caspase-9, and in turn, caspase-3. Activated caspase-3 cleaves the inhibitor of caspase-activated deoxyribonuclease (ICAD), releasing caspase-activated deoxyribonuclease (CAD) and resulting in DNA fragmentation, chromatin condensation, and other apoptotic changes [77]. Another example of the intrinsic apoptotic pathway is neuronal apoptosis induced by the withdrawal of trophic factors. In the absence of survival signals such as nerve growth factor (NGF) or brain-derived neurotrophic factor (BDNF), the JNK (c-Jun N-terminal kinase) signaling pathway becomes activated, resulting in phosphorylation and the activation of c-Jun. Activated c-Jun promotes the transcription of pro-apoptotic BH3-only proteins, including DP5/Hrk, Bim, Puma, and Bmf. These proteins activate Bax-dependent mitochondrial outer membrane permeabilization, leading to cytochrome c release into the cytosol. This pathway is therefore critically dependent on the transcriptional induction of pro-apoptotic genes following trophic factor deprivation [78] (Figure 1).

Apoptosis is governed by both internal and external pathways; however, there exist non-apoptotic pathways that are characterised by distinct morphological and mechanistic attributes, resulting in cell death. These pathways can be activated independently or serve as alternative routes when apoptosis is suppressed [79]. The non-apoptotic pathways consist of:

(a) Necroptosis: Necroptosis is a form of programmed cell death that is different from apoptosis because it breaks down plasma membranes in necroptotic cells, which causes organelles to enlarge, nuclei to condense, chromatin degradation, DNA hydrolysis, and cell lysis. TNFα is necessary for necroptosis because it binds to a receptor and activates receptor-interacting protein kinase (RIPK1) and RIPK3, which together make a necrosome complex [80]. Mitochondrial ROS induce necroptosis via enhancing RIPK1 autophosphorylation, which triggers RIPK3 recruitment and necrosome formation. RIPK3 also promotes mitochondrial energy metabolism and activates ROS generation, causing mitochondrial damage [81].

(b) Ferroptosis: Ferroptosis is an iron-dependent form of regulated cell death that is primarily driven by iron accumulation and lipid peroxidation-induced cell membrane degradation. Stressors like oxidative stress or a lack of nutrients set off this process, which lowers antioxidant levels like glutathione and leaves cells more vulnerable to damage. One important process in ferroptosis, a disorder in which cells produce excessive amounts of ROS as a result of disease or mitochondrial stress, is ROS production. Lipid peroxides are detoxified by GPX4, but excess superoxide is transformed to hydrogen peroxide during mitochondrial stress, disrupting cellular redox equilibrium and leading to ferroptosis [82].

(c) Pyroptosis: Pyroptosis is a type of defence mechanism used by the body during infections. The pyroptotic cell bursts open and releases substances like IL-1β and IL-18 that trigger the immune response. The canonical (caspase-1-dependent) pathway is triggered by signals such as pathogen-associated molecular patterns (PAMPs) or damage-associated molecular patterns (DAMPs). These signals activate a protein complex known as NLRP3 inflammasome, which in turn activates pro-caspase-1, converting it to the active form caspase-1. Activated caspase-1 processes inflammatory cytokines IL-1β and IL-18 into active forms, recruiting immune cells, and cleaves Gasdermin D (GSDMD), releasing its N-terminal fragment that forms pores in the cell membrane, causing cell swelling, membrane rupture, and pro-inflammatory contents, ultimately causing cell death [83,84]. The non-canonical pyroptosis pathway, activated by lipopolysaccharide (LPS), involves caspase-4 and caspase-5. These caspases cleave Gasdermin D, forming pores in the cell membrane, leading to cell swelling and inflammation [85,86].

(d) Parthanatos: Poly (ADP-ribose) polymerase 1 (PARP1) is a protective enzyme that helps repair DNA damage caused by mild stressors such as reactive oxygen species or calcium. It enables cells to survive and restore homeostasis. Parthanatos is a form of programmed cell death that occurs when the key enzyme PARP1 is overactivated. This overactivation causes extensive DNA damage, consumes NAD+, and hinders the cell from performing its basic functions. The release of PAR molecules that are harmful to mitochondria activates apoptosis-inducing factor (AIF), a protein that causes DNA fragmentation and chromatin condensation, which eventually lead to cell death. Parthanatos and necroptosis are mechanisms by which cells die, which involve swelling and breaking of the cell membrane. PARP1 may work with necroptosis proteins like RIPK1 and RIPK3, but further research is needed to find out more [87,88].

(e) Paraptosis: Paraptosis is a lesser-known type of PCD, where it is often linked to the activity of mitogen-activated-protein kinases (MAPKs) and it does not involve DNA fragmentation nor the formation of apoptotic cell bodies. Though the exact mechanism is still unclear, it is still unique due to its impact on the cell’s internal structures, causing swelling and the formation of large bubbles in the cytoplasm and mitochondria. It is suggested that disruptions in ion balance and protein quality management, such as calcium transfer from the ER to mitochondria, can trigger paraptosis, causing cell death. However, the exact molecular pathways remain unknown, necessitating further research [89,90].

#### 3.4.3. Crosstalk and Switching Between Cell-Death Pathways

There is cross talk between these pathways. Caspase-3, which executes apoptosis, is able to cleave gasdermin E and induce secondary pyroptosis if the cell expresses gasdermin E [91]. Caspase-8 inhibition has been shown to promote RIPK1–RIPK3-mediated necroptosis [79,84] as well as mitochondria permeabilisation causing mtDNA release and mitochondrial ROS, which can promote NLRP3 inflammasome activation and caspase-1, resulting in pyroptosis [86]. Whether a cell will undergo one of these pathways depends on the cell type, expression of executioner molecules, i.e., gasdermins and RIPK3 and the degree of stress applied [79,84].

Signals that mediate redox and nitrosative stress have also been implicated in switching between pathways. Overexpression of iNOS or nNOS in K562 cells caused pyroptosis and apoptosis. The cell fate was determined by which NOS was expressed [52]. Because this work used a leukaemic cell line, it shows that NO output can influence the choice between caspase-dependent death programmes but does not establish the same switch in neurons or glia.

In cortical neurons, glutamate-receptor-driven NOS activation triggered mitochondrial permeability transition and a transient inhibition of ATP synthesis that led to delayed apoptosis, whereas more severe insults produced early necrosis [92]. In glia, amyloid-β activates the NLRP3 inflammasome in microglia and contributes to pathology in APP/PS1 mice [93], and mitochondrial DNA release and inflammasome signalling in glial cells [94,95]. The regulated cell-death programmes that link mitochondrial injury to neuronal loss are compared in Table 1. 

### 3.5. Lipid Metabolism

Beyond their conventional role in ATP generation, mitochondria play a vital role in neurons. Lipid metabolism is an equally important yet often overlooked role of mitochondria in neurons [99]. Neuronal mitochondria help with the process of β-oxidation of fatty acids, which produces acetyl-CoA, which drives the TCA cycle for energy production. This step is especially important when glucose is limited. Fatty acids, however, cannot be used as fuel until they are transported within the mitochondrial matrix, the primary site of cellular energy production. Acyl-CoA synthase converts fatty acids into fatty acyl-CoA, their activated form required for further metabolism. The enzyme CPT1 transforms this fatty acyl-CoA into fatty acylcarnitine, which is subsequently carried across the mitochondrial membrane by certain transport proteins, regulating energy consumption according to the demands of the cell. The process involves moving fatty acylcarnitine across the mitochondrial membrane, turning it back into fatty acyl-CoA, and releasing energy as ATP by β-oxidation in a stepwise manner, thus enabling proper neuron function [100].

Dyslipidaemia is a metabolic risk factor for AD. It exacerbates inflammation and mitochondrial dysfunction through the accumulation of lipid intermediates and increased lipid peroxidation build up. This process generates reactive aldehydes that impair ETC and further contribute to oxidative stress and neuroinflammation. In addition to fatty acid imbalance, altered cholesterol metabolism also plays an important role in neurodegeneration. Cholesterol synthesis primarily occurs via the mevalonate pathway, in which acetyl-CoA is sequentially converted to HMG-CoA, mevalonate, squalene, and lanosterol. From lanosterol, cholesterol is produced by two main post-lanosterol pathways: the Kandutsch–Russell pathway and the Bloch pathway. Cholesterol is necessary for cell membrane integrity, steroid hormone synthesis, and vitamin biosynthesis [101].

In the central nervous system, cholesterol homeostasis is tightly regulated. Neurons contain elevated cholesterol levels in comparison to astrocytes and other glial cells; yet they exhibit a restricted inherent capacity for de novo cholesterol synthesis. Instead, neurons depend significantly on astrocyte-derived cholesterol. The accumulation of intermediates like lanosterol and cholesterol from the Kandutsch–Russell may reflect altered regulatory mechanisms contributing to lipid imbalance and neuronal dysfunction [100].

Cellular susceptibility is further increased by excessive cholesterol buildup in neuronal membranes, especially in mitochondrial membranes. Although ETC activity is influenced by cholesterol-enriched mitochondrial microdomains, excessive buildup causes electron transport disruption, ROS production, and mitochondrial membrane depolarisation. Such mitochondrial dysfunction is closely linked to decreased neuronal survival and neurodegenerative processes. Additionally, increased oxidised low-density lipoprotein (oxLDL) levels stimulate apoptotic pathways, cytochrome C release, and mitochondrial permeability transition pore (mPTP) opening. As a result, dysregulated lipid metabolism is directly linked to the pathophysiology of AD, since it is necessary for preserving membrane dynamics, mitochondrial integrity, and general neuronal homeostasis [102].

## 4. Mitochondrial Dynamics in Neuronal Function

Considering how closely lipid composition and cholesterol homeostasis depend on mitochondrial integrity, changes in mitochondrial lipid metabolism can have a direct impact on mitochondrial structure and function. In addition to regulating metabolism, neuronal survival depends on the dynamic remodelling of mitochondria through closely controlled processes of mitochondrial fission and fusion. The number, shape, and functional capability of mitochondria are controlled by mitochondrial dynamics, which guarantees effective energy production and cellular response to metabolic demands. These processes are critical for intracellular distribution, transmission via neuronal axons and dendrites, where energy demands are particularly high, and mitochondrial maintenance. Additionally, proper fission–fusion balance is essential for apoptotic signalling and mitochondrial quality control.

Conserved dynamin-related GTPases control these dynamic events by coordinating the splitting and merging of mitochondria to keep them working properly. When mitochondrial dynamics are disrupted, especially when there is oxidative stress and lipid dysregulation, it can lead to mitochondrial fragmentation, reduced bioenergetics, and the activation of apoptotic pathways. All these things can cause neurons to stop working properly and eventually die [25,103].

Mitochondria are highly dynamic organelles that are capable of bidirectional movement and continuous remodelling through the opposing processes of mitochondrial fission (breaking up) and fusion (forming interconnected networks). These activities, collectively known as mitochondrial dynamics, are controlled by big dynamin-related GTPases that are found in the outer mitochondrial membrane (OMM) and the inner mitochondrial membrane (IMM) [104,105]. Mitochondrial dynamics shape the structure of mitochondria, allowing them to change between spherical, tubular, and networked forms that are important for neuronal survival and adaptability. For cellular bioenergetics, calcium buffering, ROS regulation, mitochondrial DNA inheritance, mitophagy, and general neuronal survival, mitochondrial dynamics must be properly regulated [25,106].

### 4.1. Mitochondrial Fission

**Figure 2:**
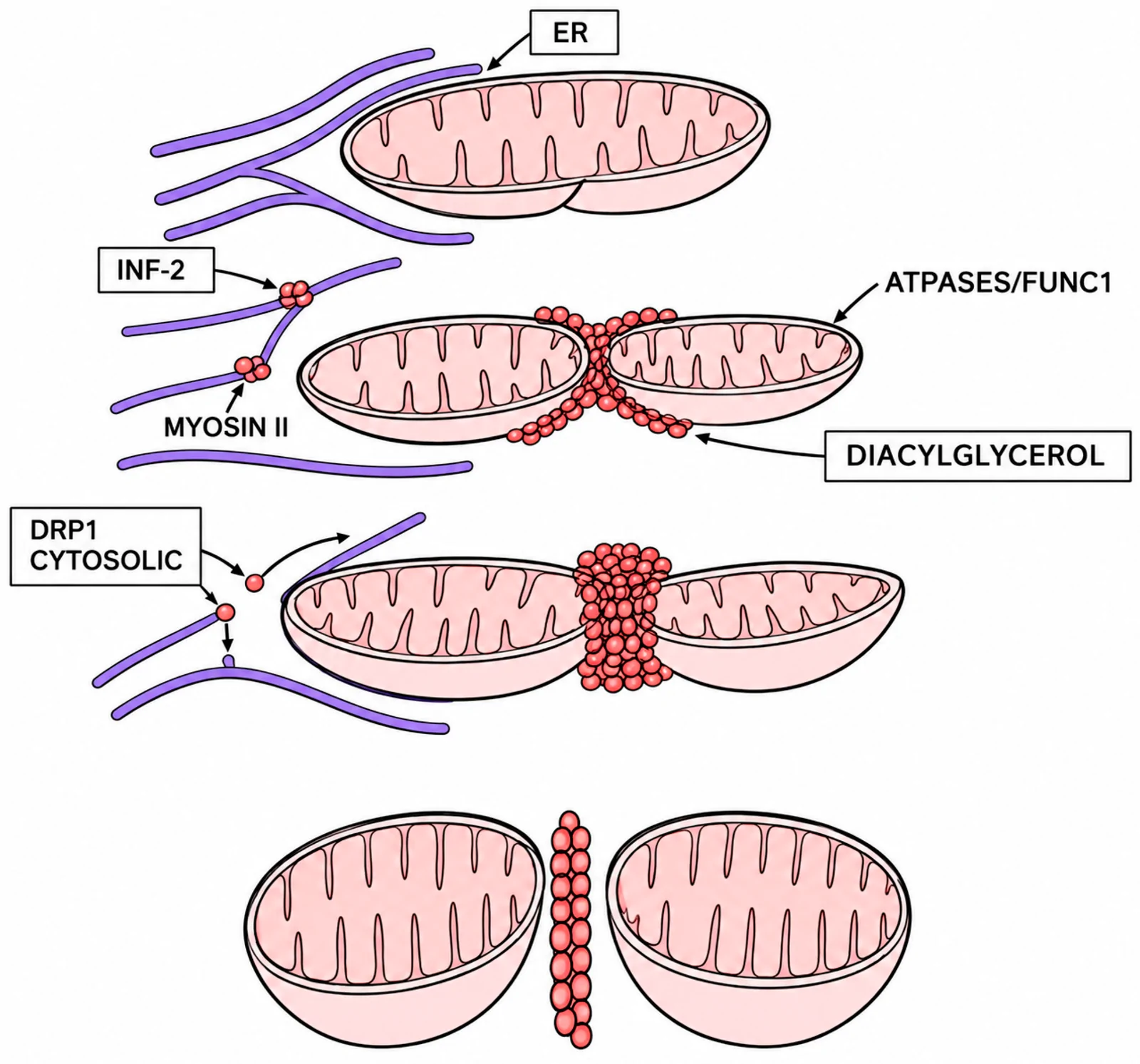
Sequential stages of endoplasmic reticulum (ER)-mediated mitochondrial fission. Mitochondrial fission is initiated when ER tubules wrap around and constrict the mitochondrion at predetermined division sites. In the first stage, inverted formin-2 (INF-2), an ER-associated actin-nucleating protein, polymerises actin filaments together with myosin II, generating the mechanical force required for initial mitochondrial constriction. In the second stage, diacylglycerol (DAG) accumulates at the constriction site together with the ATPASE/FUNDC1 complexes, facilitating further membrane remodelling. In the final stage, cytosolic dynamin-related protein 1 (DRP1) is recruited to the constriction site, where it oligomerises into a helical ring and drives GTP hydrolysis-dependent membrane scission, yielding two daughter mitochondria. The completed fission event is shown at the bottom. This sequential ER–actin–DRP1 mechanism ensures spatially precise mitochondrial division and is critically dysregulated in neurodegenerative disease.

Mitochondrial fission is a highly controlled process that enables mitochondria to split while keeping the organelle’s structure intact. Dynamin-related protein 1 (DRP1) is a cytosolic GTPase that moves to the surface of the mitochondria during division events [103]. At potential division sites, the endoplasmic reticulum (ER) envelops mitochondria, indicating the site of constriction, before DRP1 recruitment. The ER-associated protein inverted formin-2 (INF2) facilitates the production of actin filaments, with the assistance of myosin II, thereby commencing mitochondrial constriction [107,108]. Diacylglycerol (DAG)-dependent actin polymerisation significantly compresses mitochondria at ER–mitochondria interface points. Once the mitochondrion has been constricted enough, DRP1 oligomerizes around it and cuts the membrane, completing the division of the mitochondrion [109]. DRP1-driven mitochondrial fission is an initial event in apoptosis, and the suppression of excessive fission has been demonstrated to delay neuronal cell death. Controlled fission aids in the elimination of defective mitochondrial segments and enhances effective mitochondrial quality control systems [103] (Figure 2).

### 4.2. Mitochondrial Fusion

Fusion is the counterpart of the fission described in Section 4.1. Mitochondrial fusion maintains mitochondrial functionality by permitting the mixing of mitochondrial contents and the replacement of damaged components. Fusion occurs in three stages: mitochondrial tethering, fusing of the outer mitochondrial membranes (OMMs), and subsequently fusion of the inner mitochondrial membranes. This coordinated process is mediated by conserved dynamin-related GTPases, with mitofusin-1 (MFN1) and MFN2 regulating OMM fusion and optic atrophy protein-1 (OPA1) regulating IMM fusion [103].

These fusion proteins function inside an integrated molecular framework, like the yeast scaffold protein Ugo.1. The human homolog SLC25A46 has regulatory activities that promote mitochondrial division under pathological situations, resulting in mitochondrial fragmentation while maintaining interconnected mitochondrial networks during physiological states [110]. Upregulating fusion regulators like OPA1 and MFN2 has been demonstrated to reverse NMDA-induced mitochondrial fragmentation and rescue neurons from excitotoxic injury [44].

Mitochondrial membrane lipids also serve important functions in controlling fusion processes. Phosphatidylethanolamine (PE) and cardiolipin (CL), both found in mitochondrial membranes, aid in membrane fusion and regulate DRP1 activation, hence preserving structural equilibrium [111]. Phosphatidic acid (PA) contributes to membrane remodelling by boosting the conformational activation of fusion-associated proteins. These lipids lower the energetic barriers necessary for membrane curvature during both fusion and fission events. In neurodegenerative disorders, changes in lipid content tip the balance toward excessive mitochondrial fission, limiting mitophagy-mediated protection and accelerating neuronal degeneration [112] (Figure 2).

In addition to preserving somatic integrity, functional mitochondrial networks are required for dendritic development, dendritic spine formation, neurotransmitter release, long-term potentiation, and synaptic vesicle reserve pool mobilisation [113].

### 4.3. Mitochondrial Trafficking and Distribution

Mitochondrial content in dendrites, axons, and synaptic terminals is influenced by mitochondrial dynamics, trafficking, turnover, biogenesis activation, and oxidative stress [114]. Intracellular mitochondrial transport is dependent on ATP-driven motor proteins such as kinesin and dynein, as well as mitochondrial GTPases Miro1 and Miro2, and adaptor proteins TRAK1 and TRAK2 found on the OMMs.

Kinesins mediate anterograde transport to distant neuronal processes, whereas dynein motors enable retrograde transport to the soma. Rho GTPases (Miro1/2) regulate mitochondrial motility, integrity, and location within dendritic compartments in vivo [24]. Efficient trafficking ensures that mitochondria reach locations with high metabolic demand, promoting dendritic development, synaptic maintenance, and neural connection. Disruption of mitochondrial transport machinery has been linked to neurodegenerative illnesses such as PD, ALS, optic neuropathies (ON), Charcot-Marie–Tooth disease (CMT), and AD [113,114].

Mitochondria in neurons display functional heterogeneity. Those found at synapses have been shown to be more sensitive to Ca^2+^ loading compared to non-synaptic mitochondria from the same tissue [115]. Presynaptic terminals as well as dendritic and axonal mitochondria will experience varying levels of ATP and Ca^2+^ demand and are regulated by partially separate mechanisms of transport and turnover [113,114]. This division of labour implies that a defect confined to one subpopulation, for example, synaptic mitochondria, could impair synaptic function before bulk measures of mitochondrial respiration change, which is relevant to selective vulnerability.

### 4.4. Mitophagy and Mitochondrial Quality Control

Mitophagy, a specialised autophagy mechanism driven by autophagolysosomes, preferentially eliminates oxidative stress-damaged mitochondria [116]. Unlike macroautophagy, mitophagy targets defective mitochondria and is controlled by proteins such as PINK1, Parkin, LC3, and P62 [117]. Mitochondrial fission improves the segregation of damaged mitochondrial components, allowing for efficient autophagic clearance [117].

Mitophagy increases mitochondrial turnover while also activating mitochondrial biogenesis, ensuring the maintenance of functioning mitochondrial populations necessary for brain health. Mitophagy dysregulation, whether excessive or insufficient, is linked to neurite degradation and the progression of neurodegenerative disorders [117].

The PINK1–Parkin pathway is often involved in neuronal mitophagy. When the mitochondrial membrane potential drops, PINK1 builds up on the OMM. This is followed by the recruitment and activation of Parkin by ubiquitin phosphorylation. Ubiquitination of OMM proteins like VDAC by Parkin enables them to interact with LC3 and P62, which connects the ubiquitin-proteasome pathways with autophagic degradation [118,119,120,121]. Other regulators, such Optineurin, also link problems with mitophagy to neurodegenerative illnesses like PD, ALS, and HD. An alternate quality control mechanism employs mitochondrial-derived vesicles (MDVs), governed by PINK1 and Parkin, to transport oxidatively damaged proteins to lysosomes for destruction [122,123].

### 4.5. Mitochondrial Biogenesis

Mitochondrial biogenesis is a process that occurs through several coordinated steps. It involves coordinated transcription, translation, membrane synthesis, and the assembly of new mitochondria. Mitophagy and mitochondrial biogenesis work together to control how many mitochondria are in the neuronal soma and neurites. Transcriptional networks including CREB, nuclear respiratory factor-1 (NRF1), mitochondrial transcription factor A (TFAM), and PPARG coactivator-1α (PPARGC1A) control biogenesis [11,124]. Oxidative stress linked to neurodegenerative illnesses interferes with CREB signalling and diminishes PPARGC1A expression, hindering mitochondrial regeneration in models of PD, AD, and HD [125,126,127].

**Figure 3:**
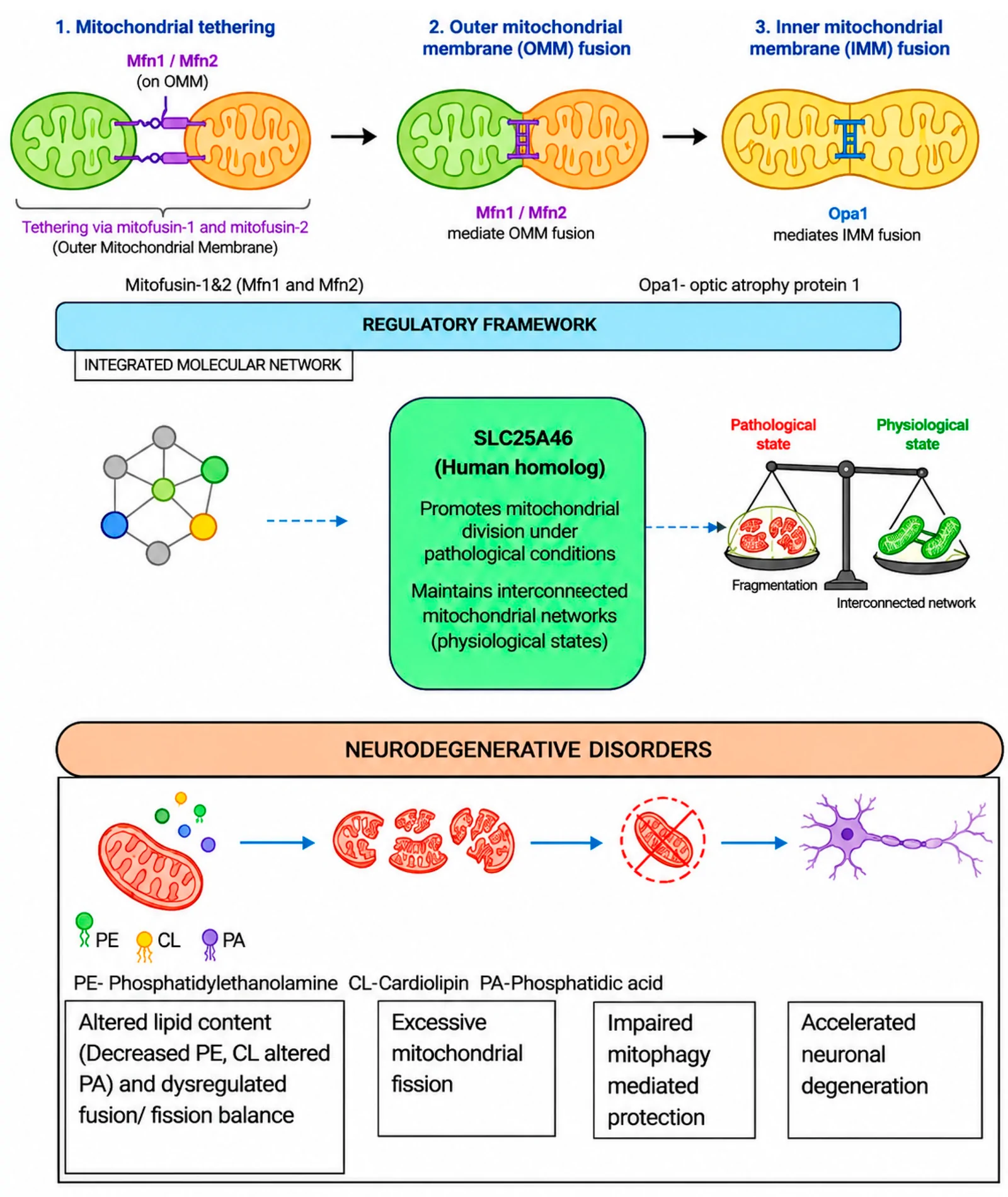
Molecular machinery of mitochondrial fusion and the consequences of fission–fusion imbalance in neurodegenerative disorders. **Upper** panel—Fusion machinery: Mitochondrial fusion proceeds in three sequential steps. First, tethering of adjacent mitochondria is mediated by mitofusin-1 (MFN1) and mitofusin-2 (MFN2) located on the outer mitochondrial membrane (OMM). Second, MFN1/MFN2 GTPase activity drives OMM fusion. Third, optic atrophy protein 1 (OPA1) mediates fusion of the inner mitochondrial membrane (IMM), restoring a continuous mitochondrial network. **Middle** panel. Regulatory framework: SLC25A46 (human homolog) functions as a context-dependent regulator: under pathological conditions, it promotes mitochondrial division, whereas under physiological conditions, it maintains interconnected mitochondrial networks. The balance between fragmentation (pathological state) and an interconnected network (physiological state) is illustrated schematically. **Lower** panel—Neurodegenerative consequences: Altered mitochondrial lipid composition—specifically decreased phosphatidylethanolamine (PE), altered cardiolipin (CL), and changes in phosphatidic acid (PA)—disrupts the fission–fusion equilibrium, leading sequentially to excessive mitochondrial fission, impaired mitophagy-mediated neuroprotection, and accelerated neuronal degeneration. Abbreviations: MFN1/2, mitofusin-1/2; OPA1, optic atrophy protein 1; PE, phosphatidylethanolamine; CL, cardiolipin; PA, phosphatidic acid; SLC25A46, solute carrier family 25 member 46.

PINK1 and Parkin also regulate mitochondrial biogenesis, with PINK1 deficiency reducing mitochondrial production and Parkin overexpression increasing it. Imbalanced mitophagy and biogenesis eventually diminish mitochondrial content in neural networks, contributing to early neurodegeneration [128]. Understanding the mechanisms governing mitochondrial shape, trafficking, and quality control is therefore critical for designing therapeutic strategies to restore mitochondrial health and prevent neurodegenerative disease development (Figure 3).

### 4.6. Mitochondrial Contacts with the ER and Lysosomes

Mitochondria interact with other organelles through contact sites. Contact sites with the ER have been identified as sites of mitochondrial fission [107,108] and facilitate the movement of Ca^2+^ and lipids between the two organelles; they are also affected at an early stage of AD [102]. Mitochondria also interact with lysosomes, and RAB7 GTP hydrolysis controls mitochondrial fission at these contact sites [129]. Vesicles generated from mitochondria, as well as mitophagy, help transport damaged mitochondrial components to lysosomes [122,123]. Because endolysosomal pathways and vesicle transport are defective in AD [130,131], and lysosomal clearance and mitophagy play a role in PD (Section 6), these contacts may be one route by which defects in proteostasis cause mitochondrial damage. Defects in lysosomal clearance have been linked to the formation of mitochondrial plaques in AD (Section 5) [132].

## 5. Mitochondrial Dysfunction in Alzheimer’s Disease

AD is the most common age-related dementia, characterised by substantial neuronal loss, mainly in the neocortex and hippocampus [133]. The disease’s histological features are extracellular amyloid beta (Aβ) plaques and intracellular neurofibrillary tangles. The buildup of these harmful proteins in the brain is strongly connected to cognitive deterioration [134]. Aβ aggregates are formed by β- and γ-secretases cleaving the amyloid precursor protein (APP) at its C-terminus, while neurofibrillary tangles are made up of hyperphosphorylated and misfolded Tau protein. Although most AD cases are sporadic, familial AD has been linked to mutations in APP or presenilin, the catalytic component of γ-secretase [134]. Amyloid plaque deposition in AD activates microglia and releases inflammatory cytokines such as IL-1β, IL-6, and TNF-α. These inflammatory mediators are hypothesised to be involved in signalling inside and between microglia and astrocytes. Persistent inflammation and compromised quality control mechanisms can lead to neurotoxicity and increased Aβ peptide synthesis. Dysfunctions in the endolysosomal system are also linked to AD development. Lysosomes and lysosome-derived structures within neurons have been found to contain Aβ peptide aggregates, including the highly lethal Aβ42 type. Transgenic AD mice have defective multivesicular bodies (MVBs), which are specialised late-stage endosomes, when Aβ42 levels are high. This malfunction affects the entire endolysosomal system, resulting in Aβ buildup in larger endocytic compartments [130]. Dysfunctional MVBs also boost APP extracellular secretion in these mice. The endosomal system is critical for intracellular APP trafficking as it contains β-secretase activity that promotes amyloid plaque development. The retromer complex facilitates the transfer of APP from endosomes to the trans-Golgi network, lowering Aβ generation. However, altered retromer complex function has been associated with AD progression, emphasising its role in the disease’s pathogenesis [131].

Mitochondrial dysfunction and oxidative stress are consistently reported in AD and are best viewed as early contributors that amplify pathogenesis. Decreases in neuronal mtDNA copy number and increased mtDNA heteroplasmy have been reported in post-mortem brains of patients with AD [135]. High-depth whole mitochondrial genome sequencing has confirmed a reduction in mtDNA content in AD patients’ brains. Furthermore, focal iron buildup has been found in various brain regions affected by AD lesions, indicating the role of redox-generated free radicals in the illness [136]. Importantly, oxidative damage to mitochondrial proteins and DNA is recognised as an early occurrence in AD, implying that it plays a role in its progression [137]. Aβ aggregates and neurofibrillary tangles disturb mitochondrial homeostasis by interacting with import machinery proteins. These interactions reduce electron transport chain (ETC) activity and enhance the generation of ROS [138,139]. Furthermore, mitochondrial dysfunction and oxidative stress have also been associated with APOE ε4, a major genetic risk factor for sporadic AD. These findings emphasize the relationship between mitochondrial failure, oxidative stress, and AD pathogenesis [140].

**Figure 4:**
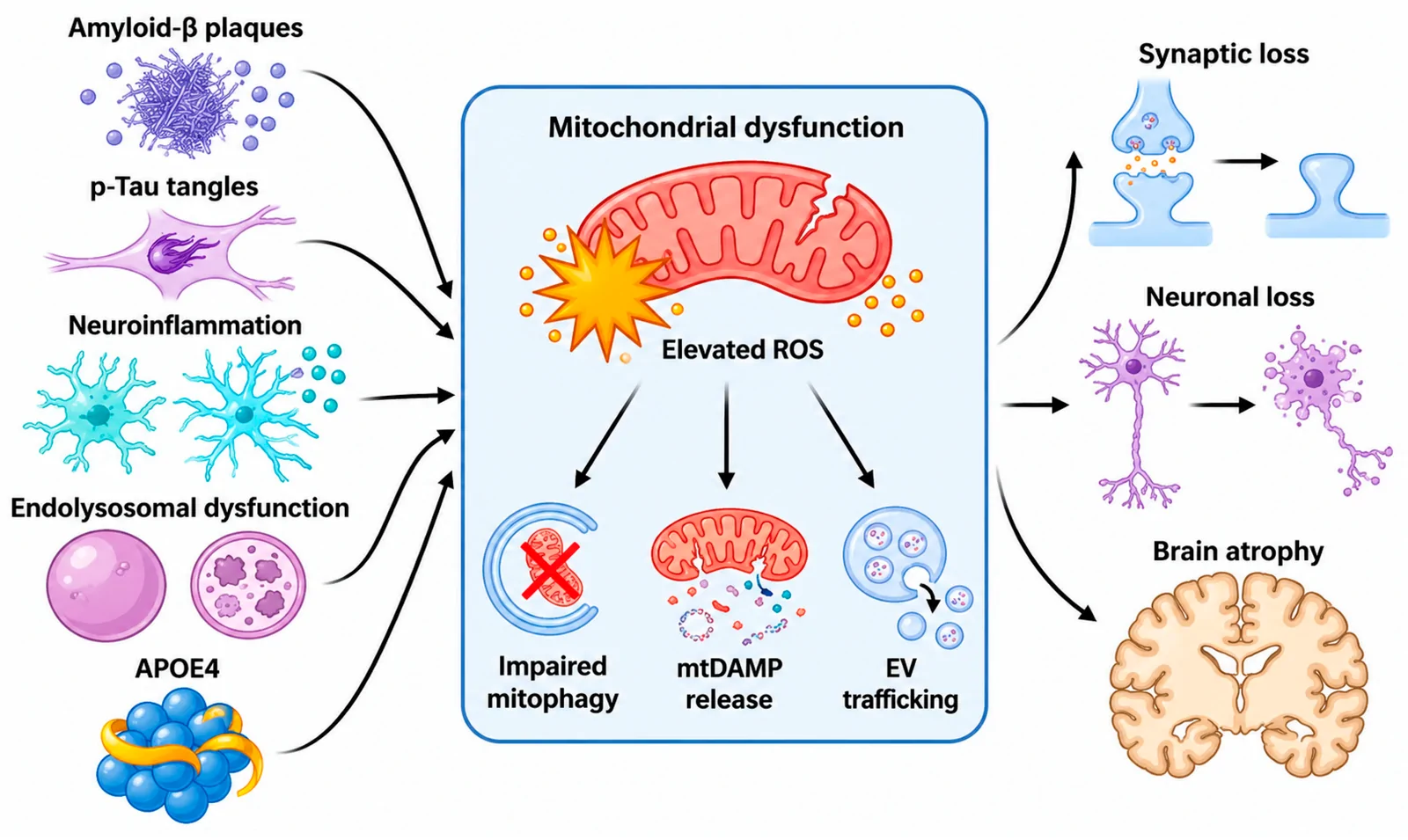
Schematic illustrating the interconnected pathways driving Alzheimer’s disease progression. Amyloid-beta plaques, hyperphosphorylated Tau tangles, neuroinflammation, endolysosomal dysfunction, and genetic risk factors (APOE4) promote mitochondrial dysfunction and increased ROS. This leads to impaired mitophagy, disrupted mitochondrial quality control, release of mitochondrial damage signals, extracellular vesicle trafficking, and ultimately neurodegeneration marked by neuronal loss, synaptic loss, and brain atrophy. Evidence tier: mitochondrial changes in AD are supported mainly by patient-tissue observations and model data (tiers ii–iii; Section 10.1). Candidate biomarkers: CSF mtDNA and EV mitochondrial cargo; candidate target: mitophagy enhancement.

Mitochondrial dysfunction in AD may result from both intrinsic organelle deficits and poor-quality control systems, including impaired mitophagy. Neuronal bioenergetic failure in this context could be linked to inflammation and neuronal death. Aβ plaque development and phosphorylated-Tau (p-Tau) buildup can lead to a cycle of impaired mitophagy and mitochondrial dysfunction, affecting neuronal function. Disruptions in the mitochondrial unfolded protein response (UPRmt) machinery may contribute to mitochondrial imbalance and Aβ proteotoxicity [141]. ATFS-1, or activating transcription factor associated with stress 1, plays an important part in this process. Depending on the health of the mitochondria, ATFS-1 can translocate into the mitochondrial matrix to decrease UPRmt or to the nucleus to begin mitochondrial turnover. Patients with AD, transgenic AD mouse models, and cultured cells treated with Aβ all show altered expression of the mitophagy receptor disrupted-in-schizophrenia 1 (DISC1). DISC1 promotes mitophagy by interacting with microtubule-associated proteins 1A/1B light chain 3, protecting synaptic plasticity against Aβ toxicity. Pharmacological therapies that restore mitophagy have been demonstrated to improve cognitive performance and reduce Aβ proteinopathy in APP/PS1 mouse models, supporting a contribution of defective mitophagy to disease progression in these models. These therapies have also been shown to diminish Tau phosphorylation and alleviate microglia-induced inflammation [142,143]. Mitophagy malfunction and subsequent mitochondrial impairment in AD may cause the release of damaged mitochondrial components, activating innate immune responses. Mitochondrial damage-associated molecular patterns (DAMPs) have been found in extracellular vesicles (EVs) in age-related illnesses, including neurodegenerative disorders (Figure 4). If this pathway is active in AD, it may explain the development of systemic inflammation associated with mitochondrial dysfunction. Analysing EV cargo may shed light on how Aβ42, T-Tau, P-Tau, and neurofilament light polypeptide circulate in AD [144,145].

Mitochondrial abnormalities can be thought of as being primary or secondary to AD. There are several pieces of evidence that suggest mitochondrial changes occur before proteinopathy and others that occur after. Decreased mtDNA copy number, heteroplasmy and oxidation of mitochondrial proteins/DNA have been found in patients’ brains and oxidation occurs at an earlier stage [135,137] (tier ii observational); however, we are unable to determine if this occurs before amyloid/tau using postmortem samples. There is also evidence for defective mitophagy, UPRmt and downregulation of the ETC by Aβ and tau in mice, worms and cells [138,139,141,142,143] (tier iii), where the buildup of Aβ or tau is what was initiated. Mitochondrial genes do not cause classical AD. Based on current evidence, it is more likely that mitochondrial dysfunction arises early in the course of the proteinopathy and then feeds back into it, but it cannot be said to be causative with certainty. More human data with biomarkers that track progression will be required to test these events in order.

Mitochondrial plaques. Mitochondrial plaques have recently been found in the brains of APP/PSEN1 mice using a mitophagy reporter (mt-Keima) and in humans with AD. However, they were not found in normal brains [132]. Mitochondrial plaques are made up of clusters of mitochondria inside and/or outside of lysosomes in dystrophic neurites. They result from deficient mitophagy and dysfunctional lysosomes and can form without amyloid plaques in earlier stages and can fuse with amyloid plaques to create hybrid plaques [132]. This is a recent finding, derived mostly in mouse models and some human brains. It needs to be further studied in humans to determine whether it plays a role in AD pathology.

## 6. Mitochondrial Dysfunction in Parkinson’s Disease

The mitochondrial theory of PD posits that disturbances in oxidative phosphorylation, mitochondrial biogenesis, or mitophagy are implicated in PD-related symptoms [11]. Langston originally suggested this link in 1983 after observing that intravenous drug users quickly developed Parkinsonian symptoms after being exposed to 1-methyl-4-phenyl-1,2,3,6-tetrahydropyridine (MPTP). 1-Methyl-4-phenylpyridinium (MPP+), a by-product of MPTP, inhibits mitochondrial complex I activity. In the central nervous system, MPP+ is absorbed via the dopamine active transporter, which causes dopaminergic neurons in the substantia nigra pars compacta (SNpc) to die off selectively. This occurrence has been replicated in MPTP-treated mice and nonhuman primates, rendering these models extensively utilised for PD research [146,147]. Similar results were noted with rotenone, a pesticide that similarly obstructs mitochondrial complex I. Rotenone spreads throughout the central nervous system, but its greatest harmful effects are shown in SNpc dopaminergic neurons. This supports the hypothesis that neurons that are highly sensitive to changes in energy are the ones most affected. Epidemiological studies further substantiate this, revealing that agricultural workers exposed to rotenone exhibited an elevated chance of acquiring PD [148,149].

**Figure 5:**
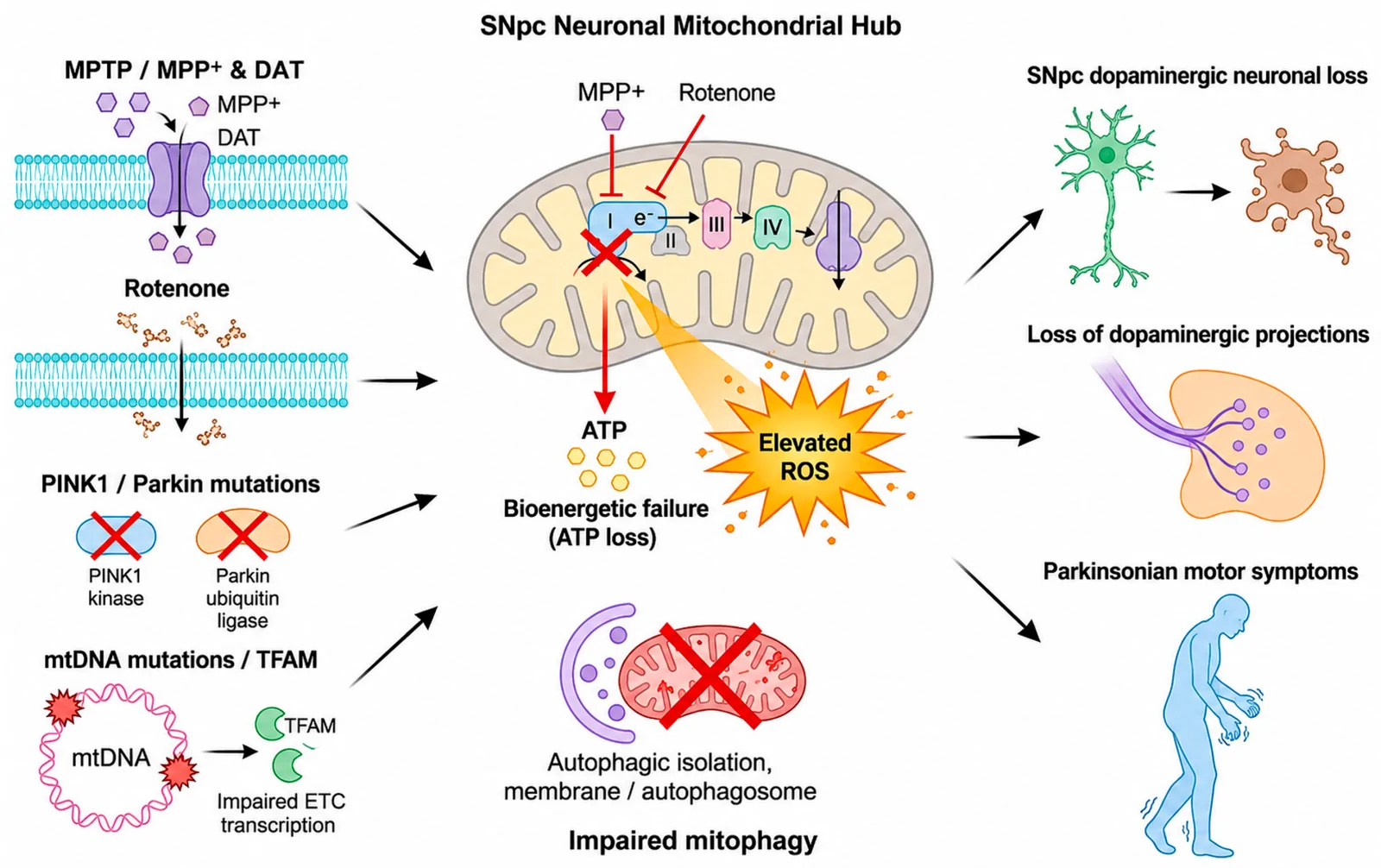
Schematic overview of the major mechanisms underlying Parkinson’s disease progression. Hallmarks of Parkinson’s disease, environmental toxins, and genetic risk factors contribute to mitochondrial dysfunction and oxidative stress. These changes impair mitophagy, mitochondrial quality control, and cellular bioenergetics, leading to selective neuronal vulnerability, progressive dopaminergic neuronal loss, and the motor symptoms characteristic of Parkinson’s disease. Evidence tier: PINK1/Parkin dysfunction is supported by human genetics (tier i). Candidate biomarkers: mitochondrial damage-associated inflammatory markers and CSF mtDNA; candidate target: USP30 inhibition.

Genetic mutations in PD patients strengthen the link between mitochondrial dysfunction and the disease. Mutations in the genes PINK1 and PRKN (previously PARK2), which encode the mitophagy-related proteins PINK1 and Parkin, are associated with autosomal recessive early-onset PD [150,151]. The similarities in clinical features between Parkin-related and PINK1-related early-onset PD suggest that mitochondrial quality control systems, rather than specific mutations, play an important role in PD pathogenesis. Furthermore, somatic mutations in mitochondrial DNA (mtDNA) have been found in sporadic patients with PD, with their frequency increasing with age, reflecting the increased incidence of PD in older people. Since the function of numerous electron transport chain (ETC) proteins is dependent on coordinated transcription from both nuclear and mitochondrial DNA, an accumulation of mtDNA mutations is likely to impede mitochondrial oxidative phosphorylation [152]. Post-mortem investigations in older people show that mtDNA mutations are more common in the substantia nigra pars compacta (SNpc) than in other brain regions, regardless of PD diagnosis [153,154]. Although the reason for the selective accumulation of mtDNA mutations in SNpc neurons is unclear, these findings point to a relationship between ageing, PD, and mitochondrial bioenergetic failure. This pathogenic cascade is thought to begin with age-related genomic instability, which leads to mitochondrial dysfunction, followed by bioenergetic failure in vulnerable neuronal populations, and finally neuronal death and the emergence of PD symptoms [11]. The MitoPark animal model, which conditionally knocks out mitochondrial transcription factor A (TFAM) in dopaminergic neurons, provides additional evidence for this concept. While these mice are born healthy, they suffer SNpc dopaminergic neuronal degeneration and motor impairments throughout ageing, which is like how PD progresses in people (Figure 5). Collectively, this evidence highlights mitochondrial dysfunction as a key factor in the pathogenesis of PD, identifying the condition as one caused by bioenergetic failure [155,156,157].

There is evidence to suggest that mitochondrial dysfunction plays a tier i role in PD: mutations that result in loss of function for PINK1 and PRKN have been linked to autosomal recessive forms of parkinsonism [150,151]. Firstly, the PINK1/Parkin pathway may only be involved when there is some level of stress on the mitochondria. This is because mitophagy under basal conditions in vivo does not require PINK1 [158]. Secondly, most patients will have the sporadic form of the disease where mitochondrial dysfunction could be due to aging, environmental factors, α-synuclein and nitrosative modifications to Parkin and PINK1 [159,160] rather than mutations.

## 7. Mitochondrial Dysfunction in Amyotrophic Lateral Sclerosis

Pathogenic variants in ALS-associated nuclear genes, including FUS, SOD1, and CHCHD10, can disrupt mitochondrial homeostasis [159]. The SOD1 gene is critical for maintaining mitochondrial homeostasis and avoiding apoptosis. In its mutant form, SOD1-G93A, the gene interacts with Bcl-2, forming a mitochondrial complex. Bcl-2 prevents apoptosis by controlling the release of cytochrome C and mitochondrial caspase activation [160]. The C9orf72 gene, which codes for a mitochondrial inner membrane-associated protein, is a prevalent hereditary cause of ALS due to GGGGCC sequence repeat expansions. This mutation causes dipeptide repeat (DPR) proteins to accumulate in mitochondria, resulting in neuronal aggregates. C9orf72 also helps to stabilise TIMMDC1, a crucial component of mitochondrial complex I assembly. Loss of C9orf72 function or haploinsufficiency lowers complex I activity, as demonstrated in neurons from patients with C9orf72-ALS, which contributes to disease progression [161,162]. Mitochondrial failure in ALS has been linked to oxidative stress [163]. Reduced mitochondrial activity has been reported in fibroblasts carrying TARDBP (TDP-43) and C9orf72 mutations [164]. In addition, increased expression of the fission proteins FIS1 and DRP1, together with a progressive decrease in the fusion proteins MFN1 and OPA1, has been described from early symptomatic stages in a transgenic mouse model of ALS [165]. These findings emphasise the role of mitochondrial dynamics in ALS. Excessive mitochondrial fragmentation can result from fusion or fission processes, inducing apoptosis and affecting mitochondrial functions such as the electron transport chain (ETC), calcium homeostasis, ATP synthesis, and the creation of ROS. This mitochondrial dysfunction is important to the pathogenesis of ALS [165,166].

Mitophagy promotes cell development by effectively eliminating ageing and damaged mitochondria. The process begins with the breakdown of the mitochondrial tubular network and the isolation of damaged mitochondria in response to internal and external stimuli. Surface receptors then target these mitochondria, forming an isolation membrane that wraps them within autophagosomes [117]. The autophagosomes are then transported and merged with lysosomes, which release enzymes that destroy mitochondria. The deteriorated components are then recycled for cellular applications. Optineurin (OPTN) is a critical receptor in the PINK1/Parkin-dependent mitophagy pathway. It attaches ubiquitin chains on damaged mitochondria to aid in their clearance. This pathway is dependent on both OPTN and its related kinase, TBK1. The dysfunction or deletion of OPTN or TBK1 hampers mitophagy, resulting in the buildup of damaged mitochondria. The autophagy receptor SQSTM1/p62 also contributes by connecting with LC3 via its LC3-interacting region (LIR), which aids in the destruction of ubiquitinated molecules. However, the ALS-linked L341V mutation in SQSTM1 impairs its capacity to recognise LC3B, rendering it less efficient at integrating into autophagic vesicles. The loss of SQSTM1 function affects the autophagy–lysosomal degradation pathway, whereas the L341V mutation lowers SQSTM1’s affinity for LC3, delaying degradation and further deregulating the autophagic process [167,168].

**Figure 6:**
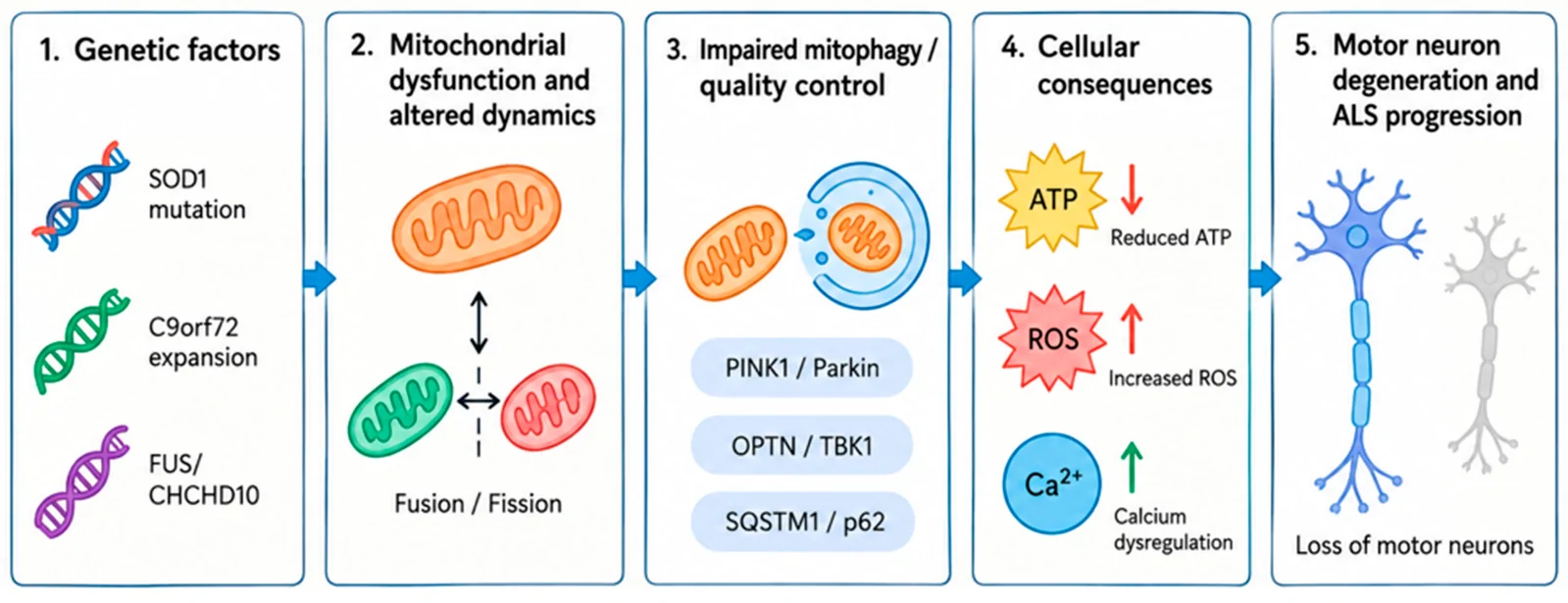
Mitochondrial dysfunction and impaired quality control pathways in amyotrophic lateral sclerosis (ALS). Evidence tier: CHCHD10 variants are supported by human genetics (tier i); C9orf72, SOD1 and TDP-43 effects derive from patient cells and models (tiers ii–iii). Genetic mutations in ALS-associated genes, including SOD1, C9orf72, FUS, and CHCHD10, disrupt mitochondrial homeostasis through impaired apoptosis regulation, mitochondrial complex I dysfunction, and abnormal protein accumulation. These alterations promote mitochondrial fragmentation, imbalance of mitochondrial dynamics, oxidative stress, reduced ATP production, and disturbed calcium regulation. Defects in mitophagy pathways involving PINK1/Parkin, OPTN, TBK1, and SQSTM1/p62 impair the removal of damaged mitochondria, leading to their accumulation and further cellular stress. Collectively, mitochondrial morphological abnormalities, impaired energy metabolism, and defective mitochondrial quality control contribute to progressive motor neuron degeneration and ALS pathogenesis.

Mitochondrial dysfunction can be caused by aberrant mitochondrial morphology. According to research, C9orf72 helps regulate energy homeostasis by stabilising mitochondrial complex I. Loss of C9orf72 destabilises complex I, which contributes to motor neuron degeneration. Interestingly, preserving normal mitochondrial shape appears to protect C9orf72 from destruction. Alterations in mitochondrial shape in ALS cells were associated with lower mitochondrial membrane potential and poor respiratory chain complex function [104]. In the SOD1-G93A transgenic mouse model and various cell types, poor mitochondrial shape was associated with decreased ATP generation, disturbed Ca^2+^ regulation, and higher ROS levels (Figure 6). These findings show that morphological abnormalities in mitochondria can directly or indirectly impair mitochondrial function, providing compelling evidence for the role of mitochondrial dysfunction in ALS aetiology [105,166].

Mitochondrial dysfunction has also been shown with other ALS linked proteins. TDP-43 is known to aggregate in ALS patients and can be found localised to mitochondria. Preventing TDP-43 from localising to mitochondria showed decreased toxicity in cell and in vivo models [169]. For C9orf72-ALS/FTD the dipeptide repeat protein poly(GR) was shown to cause mitochondrial dysfunction, leading to increased oxidative stress and DNA damage in iPSC motor neurons derived from patients [170]. Variants of CHCHD10 have been directly linked to ALS/FTD through cristae organisation and respiration.

There are currently no biomarkers for mitochondria that can be utilized in ALS. The only form of therapy we can target based on genotyping is Tofersen. In a phase 3 trial, this drug lowered the levels of SOD1 and neurofilaments, but did not show efficacy on a clinical outcome at 28 weeks [171]. This treatment does not directly target mitochondria but could help identify patients with these mutations. There is a major role of mitochondrial dysfunction in ALS/FTD caused by mutations in mitochondrial genes and may also play a role in sporadic ALS.

## 8. Mitochondrial Dysfunction in Huntington’s Disease

HD is a genetic neurological condition caused by the amplification of a CAG triplet repeat in the huntingtin (HTT) gene, which results in protein misfolding and aggregation. The condition causes progressive behavioural and motor deficits, as well as a cognitive decline [172]. Striatal GABAergic medium spiny neurons (MSNs) are especially vulnerable in HD. The gradual dysfunction of MSNs is closely related to the development and severity of motor complaints. A distinguishing aspect of HD is altered mitochondrial morphology, which varies by cell type. In peripheral tissues such as lymphoblasts, myoblasts, and fibroblasts, mitochondria have an expanded shape, whereas neurons have greater mitochondrial fragmentation [173]. These abnormalities are linked to mitochondrial dysfunction in all HD cells, including decreased electron transport chain activity, impaired oxygen consumption, disturbed Ca^2+^ buffering, and poorer ATP and NAD^+^ synthesis. It has been hypothesised that mitochondrial defects produced by mutant HTT (mHTT) have a substantial influence on MSNs due to their high energy demands, making them more vulnerable to HD [174]. Supporting this hypothesis, mitochondria isolated from the striatum of adult rats were more sensitive to Ca^2+^-induced membrane permeabilization than those from the cerebral cortex, indicating a higher susceptibility to metabolic stress. Additional factors contributing to this cell-specific pathology include cell-type-specific processing or localization of mHTT, abnormal interactions between mHTT and proteins specific to brain regions, and tissue-specific differences in CAG repeat instability. These processes play important roles in promoting MSN degradation and aggravating mitochondrial stress in HD [175].

**Figure 7:**
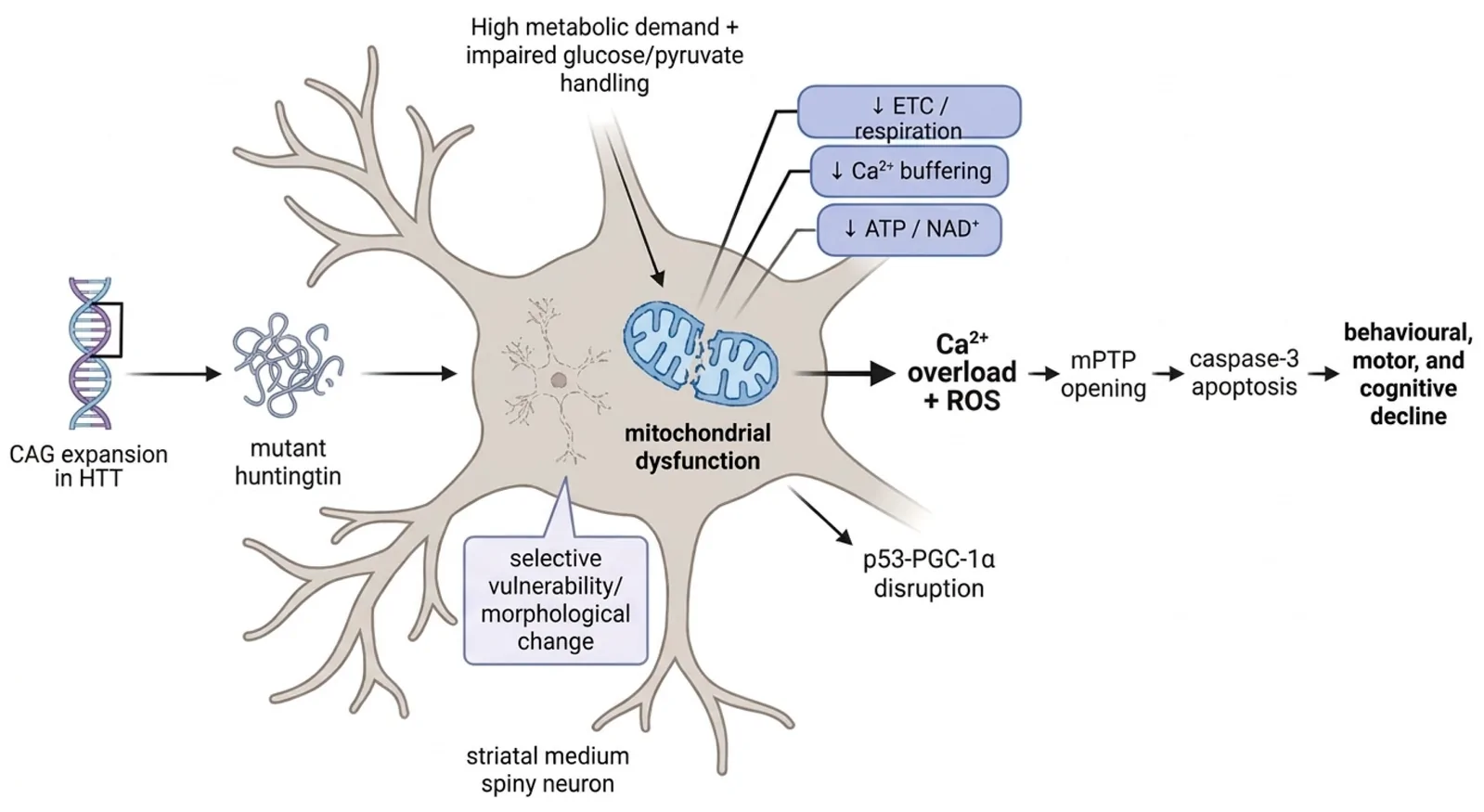
Mitochondrial dysfunction driving Huntington’s disease (HD) pathology. (1) CAG repeat expansion in HTT produces mutant huntingtin, causing selective vulnerability and morphological changes in striatal medium spiny neurons (MSNs). (2) This drives mitochondrial dysfunction—fragmentation in neurons, expanded morphology in peripheral cells—with reduced ETC activity, impaired respiration, disturbed Ca^2+^ buffering, and low ATP/NAD^+^. (3) High metabolic demand and early glucose/pyruvate handling deficits worsen mitochondrial defects, triggering Ca^2+^ overload, ROS production, and mPTP opening. This activates caspase-3-mediated apoptosis and disrupts p53–PGC-1α transcriptional signalling, further impairing mitochondrial biogenesis. Together, these pathways drive progressive behavioural, motor, and cognitive decline in HD. Evidence tier: mutant-huntingtin-related mitochondrial defects are supported by patient imaging and tissue and by models (tiers ii–iii) and are interpreted as an early amplifier of striatal degeneration; blood neurofilament light chain is the comparator biomarker.

Patients with HD and HD mouse models have well-documented metabolic impairments. Presymptomatic patients with HD using positron emission tomography (PET) and proton nuclear magnetic resonance (^1^H-NMR) showed lower striatal glucose uptake and pyruvate utilisation years before motor symptoms appeared. These findings show that mitochondrial dysfunction may have an early role in disease progression [176]. However, research in HD mouse models has shown that alterations in MSN dendritic structures occur prior to detectable mitochondrial respiratory defects, indicating that energy deficits may arise as a consequence of neuropathological changes. Whether mitochondrial dysfunction is the source or effect of disease pathology, the available data are most consistent with an early amplifying role in the course of HD [177,178]. In recent years, there has been a substantial focus on developing therapeutic approaches aimed at improving mitochondrial activity. These techniques include enhancing ATP generation, decreasing mitochondrial membrane permeability, and mitigating oxidative damage [5,13]. Mitochondria also play an important role in controlling cell death by acting as a reservoir for pro-apoptotic chemicals. Dysregulated mitochondrial activity, Ca^2+^ excess, and ROS buildup can open the mPTP. This event activates the intrinsic apoptotic pathway, which is defined by outer mitochondrial membrane permeabilization, cytochrome c release, and caspase-3 activation [179,180]. Extensive research has focused on p53 and PGC-1α transcription factors in HD, as they play a role in mitochondrial malfunction, apoptosis, and neurodegeneration. Their dysregulation contributes significantly to these pathogenic processes in HD (Figure 7) [127,181]. 

There are many direct effects on mitochondria caused by mutant huntingtin. It can downregulate PGC-1α and downstream targets [127] and interacts with fission GTPase DRP1, leading to increased activity and over fission, causing damage to neurons in HD models and human samples [182]. Blood neurofilament light chain is elevated in HD as a function of disease load and can predict progression of the disease [183]. There is currently no validated biomarker for mitochondria. Mitochondria-directed treatment has not succeeded in HD, since high-dose co-enzyme Q10 did not slow functional decline in the 2CARE trial [184].

In summary, mutant huntingtin causes damage to cells. Hypometabolism in the striatum is observed prior to motor symptoms [176], however, alterations in dendrites of MSNs occur before respiratory deficits in mice [177,178], suggesting that mitochondrial dysfunction may be a contributing factor to early stages of striatal cell death. A comparative summary of mitochondrial pathology across all four diseases is presented in Table 2.

## 9. Therapeutic Strategies Targeting Mitochondria

### 9.1. Mitochondria-Targeted Antioxidants

Antioxidant therapies have gained popularity due to their ability to restore mitochondrial activity and reverse neuronal damage. Traditional antioxidants, on the other hand, frequently fail to target damaged mitochondria, resulting in potentially detrimental systemic effects. In recent decades, innovative mitochondria-targeted antioxidants (MTAs) have been produced and tested in both in vitro and in vivo research. Key agents are compared in Table 3. These compounds are designed to alleviate mitochondrial oxidative stress, boost energy production, and restore mitochondrial membrane potential in neurons [229]. This review explores the therapeutic potential of four thoroughly investigated MTAs: MitoQ, SkQ1, MitoVitE, and MitoTEMPO. These compounds are classified as MTAs conjugated with lipophilic cations, which allow them to aggregate preferentially in mitochondria. Among these cations, triphenylphosphonium (TPP) has long been utilised as a carrier for antioxidant compounds, particularly hydrophobic ones that would otherwise struggle to permeate cellular membranes and reach mitochondria [230]. TPP, like other lipophilic cations, successfully targets mitochondria thanks to its positive charge, which is drawn to the mitochondrial inner membrane’s high negative transmembrane potential. The electrochemical gradient between the cellular membrane (−30 to −60 mV) and the inner mitochondrial membrane (−150 to −180 mV) promotes the selective accumulation of TPP-based compounds in mitochondria while minimising off-target effects. Once within the cell, the TPP moiety aids the transport of these compounds into the mitochondrial matrix. Within mitochondria, the TPP-antioxidant conjugate is found in the inner layer of the mitochondrial inner membrane, with the hydrophobic functional group embedded among the lipid tails and the polar TPP head facing the matrix. The stability of this location is determined by the size and hydrophobicity of the functional group [231]. Several antioxidants have been conjugated with TPP over the years to improve mitochondrial absorption, including MitoPBN, MitoPeroxidase, SkQR1, MitoQ, SkQ1, MitoVitE, and MitoTEMPO. This review focuses on MitoQ, SkQ1, MitoVitE, and MitoTEMPO since they are widely used and evaluated in cellular and preclinical models of neurodegenerative disorders [232].

#### 9.1.1. Mitoquinone (MitoQ)

Mitoquinone (MitoQ) is a modified ubiquinone conjugated with the TPP carrier that has become one of the most intensively researched mitochondria-targeted antioxidants (MTAs) in the last decade [233]. MitoQ has been shown to be useful in animal models for a variety of ailments, including kidney disease, metabolic syndromes, systemic inflammatory response syndrome, cardiovascular disease, eye disorders, arthritis, and age-related conditions like neurodegenerative disease. MitoQ, like another TPP-linked antioxidant, SkQ1, has demonstrated neuroprotective and anti-aging activities [12]. Encouraged by its promising results in several disease models, researchers began clinical trials to investigate MitoQ’s potential advantages for neurodegenerative diseases. However, clinical trials for serious diseases have not been able to replicate the results observed in in vivo models. Despite this, MitoQ is currently accessible as a dietary supplement, with studies confirming its safety and acceptability when consumed orally [234].

MitoQ’s antioxidant activity is linked to its capacity to neutralise ROS, such as superoxide, peroxyl, and peroxynitrite, once they enter the mitochondria. MitoQ has the unusual ability to be recycled; its oxidised form can be regenerated into its active ubiquinol form by the electron transport chain, allowing it to remain active in the mitochondria. This property allows for a stable concentration to be maintained with continuous low-dose administration [235]. Preliminary investigations in mice revealed a tolerable dose of 20 mg/kg body weight, with considerable toxicity at 27 mg/kg. However, long-term, low-dose MitoQ therapy in mice has demonstrated no toxicity, implying that toxic effects are caused by the excessive accumulation of TPP in mitochondria, which disturbs their normal function [12]. MitoQ has been shown to protect against PD in in vitro models. In PD cell lines, MitoQ therapy reduced mitochondrial fragmentation as well as Bax activation and translocation [236]. Another study assessed the impact of MitoQ on both cellular and animal models simultaneously. In animal experiments, MitoQ decreased toxicity, increased mitochondrial membrane potential, and decreased apoptotic marker expression [237]. MitoQ therapy inhibited tyrosine hydroxylase inactivation, mitochondrial potential loss, and caspase-3 activation while increasing motor performance in the mice. These promising findings prompted therapeutic trials in patients with PD. Unfortunately, in a 12-month, double-blind, placebo-controlled phase 2 trial in early PD (n = 128; 40 or 80 mg/day), MitoQ did not slow disease progression, even at the highest dose [238,239]. This lack of efficacy could be due to limited brain penetration or to the severity of neuronal injury in patients at the time of therapy beginning.

MitoQ has also been studied in models of AD, both in vitro and in vivo. In vitro studies demonstrated that MitoQ therapy lowered hydrogen peroxide levels, enhanced ATP generation, and improved mitochondrial membrane potential [240]. Two in vivo studies used a transgenic mouse model of AD that expressed three human mutant genes: amyloid precursor protein (APP), presenilin-1 (PS-1), and four-repeat tau. APP and PS-1 are linked to early-onset AD in humans, whereas tau mutations are linked to frontotemporal dementia. MitoQ had no effect on wild-type mice, but it improved behavioural and cognitive function in a transgenic AD model. The isolated brains showed decreased lipid peroxidation, Aβ buildup and toxicity, caspase activation, and synaptic loss [240,241]. Researchers delivered MitoQ to a transgenic mouse model of ALS and observed drug buildup and disease development in the brain, spinal cord, and muscle tissues. Muscle tissue was included in the analysis because ALS causes mitochondrial failure in muscles, making it an important sample for studying mitochondrial structural modifications. High-resolution respirometry revealed that MitoQ accumulated in the spinal cord and quadriceps muscle, delaying the loss in muscle function. MitoQ therapy dramatically reduced oxidative stress as well as disease-related pathological characteristics. In transgenic animal models, mitochondria-targeted antioxidants like MitoQ have demonstrated enhancement of neuromuscular performance and a substantial increase in lifespan, underscoring their therapeutic potential in neurodegenerative disorders. For instance, long-term MitoQ treatment in older transgenic mice led to a longer lifespan and better functional outcomes than in untreated controls. This supports the idea that changing the levels of oxidative stress in mitochondria can prevent the progression of disease [241,242].

#### 9.1.2. SkQ1

SkQ1, like MitoQ, has antioxidant properties, however, it is made of the mitochondria-targeting chemical TPP conjugated with plastoquinone rather than ubiquinone. Plastoquinone, like ubiquinone, can neutralise superoxide in the mitochondria. SkQ1 improved mitochondrial activity and cellular respiration in an mtDNA mutator mouse model, resulting in normalised weight, increased mobility, delayed age-related diseases, and a longer lifetime [243,244]. Following these findings, Visomitin, a SkQ1-based formulation initially developed in Russia for ophthalmic use, has been evaluated in dry-eye disease: the U.S. phase 2b/3 trial VISTA-1 did not meet its nominal co-primary endpoints, whereas the sponsor reported VISTA-2 as positive on a prespecified key secondary endpoint (central corneal staining) [245,246]. Both trials are registered with results on ClinicalTrials.gov [247,248], and we did not identify a full peer-reviewed report of VISTA-2. These are ocular indications and are relevant here only because they provide human tolerability data for a TPP^+^–plastoquinone compound; they do not demonstrate efficacy in neurodegenerative disease [249]. Experimental evidence substantiates the therapeutic significance of SkQ1 relative to other antioxidants. In cardiomyoblast (H9c2) models, mitochondria-targeted antioxidants such as SkQ1 and mitoquinone (MitoQ) exhibited markedly superior protective benefits compared to traditional antioxidants like vitamin C. In particular, pretreatment with these triphenylphosphonium (TPP)-conjugated compounds significantly diminished mitochondrial ROS levels, maintained mitochondrial integrity, and improved cell survival after oxidative stress [250]. This enhanced efficacy is due to their capacity to selectively accumulate in mitochondria, facilitating the targeted neutralisation of ROS at the principal site of production [233]. These findings collectively highlight the superiority of mitochondria-targeted antioxidants compared to non-targeted antioxidants and endorse their potential efficacy in alleviating oxidative damage in cellular disease models.

#### 9.1.3. MitoVitE

MitoVitE, also known as Mito-tocopherol, is a type of Vitamin E that targets the mitochondria. The TPP molecule is combined with α-tocopherol, a strong fat-soluble antioxidant found naturally. MitoVitE, like other TPP-linked antioxidants, is rapidly absorbed by cells and accumulates preferentially in mitochondria due to its hydrophobic nature. Initial in vivo studies demonstrated that MitoVitE is found in mitochondria-rich tissues such as the heart, brain, muscles, liver, and kidneys, all of which are highly susceptible to oxidative stress [251]. Early studies showed that MitoVitE successfully combats lipid peroxidation, preserving mitochondria and cells from oxidative damage, including those without mitochondrial DNA [252]. Further in vitro research has confirmed similar findings [253]. For example, one study found that MitoVitE reduces caspase activation and death in bovine aortic endothelial cells after peroxide exposure while retaining mitochondrial membrane potential [254]. Another study found that MitoVitE outperformed non-mitochondrial-targeted antioxidants in shielding cells from endogenous oxidative stress and death in cultured fibroblasts originating from Friedreich’s ataxia [255]. However, despite its established efficacy in mitigating oxidative damage, MitoVitE’s potential application in treating neurodegenerative illnesses has yet to be completely studied.

#### 9.1.4. MitoTEMPO

MitoTEMPO is a mitochondria-targeted antioxidant (MTA) made up of (2,2,6,6-Tetramethylpiperidin-1-yl)oxyl (TEMPO, a piperidine nitroxide) and TPP. MitoTEMPOL is a related chemical that has the nitroxide radical TEMPOL (4-hydroxy-2,2,6,6-tetramethyl-piperidine-1-oxyl). Both molecules are stable and have an aminoxyl radical, which lets them neutralise mitochondrial superoxide. This mechanism changes them into hydroxylamine, which is a radical scavenger that works like a superoxide dismutase (SOD) to convert superoxide to hydrogen peroxide, which is subsequently detoxified by catalase and glutathione peroxidase. MitoTEMPOL has been demonstrated to have antioxidant effects in vitro by reducing oxidative stress and mitochondrial dysfunction in rat liver extracts. However, its possible significance in neurodegenerative illnesses has not yet been studied [256].

However, MitoTEMPO has been thoroughly investigated in both in vitro and in vivo models of neurodegenerative illnesses, such as PD and AD. Researchers observed that oxidative stress in neurons causes more hydrogen peroxide (H2O2) to be generated in PD models. MitoTEMPO significantly decreased H2O2 levels and inhibited cell death, underscoring its function in mitigating redox imbalance in neurodegeneration [257]. A study on the enzyme isocitrate dehydrogenase (IDH2), which controls redox balance and oxidative damage, showed that silencing IDH2 in N2a cells and knockout mice subjected to MPTP caused more oxidative stress and neuron death. Giving MitoTEMPO to these animals increased the number of neurons that expressed tyrosine hydroxylase, which protected the neurons [258]. In AD models, primary mouse cortical neurons subjected to amyloid β (Aβ) demonstrated oxidative damage, characterised by elevated ROS generation and lipid peroxidation. MitoTEMPO reduced these effects, prevented SOD2 inhibition, and protected mtDNA from destruction. Another study looked into tau protein clumping in the hippocampus of human brains with AD, tauopathy mice, and neurons from tauopathy animal models. Tau aggregates were linked to higher levels of ROS, especially in the hippocampus. When tauopathy neurons were treated with MitoTEMPO, tau oligomer levels decreased markedly, approaching those of the control samples. MitoTEMPO also enhanced oxidative phosphorylation, diminished mitochondrial abnormalities, and decreased ROS levels. Researchers have also tested MitoTEMPO on mice with the Wobbler mutation, which is similar to ALS pathophysiology. Tissue samples from these animals exhibited increased p53 and ROS levels, coupled with reduced glutathione levels. MitoTEMPO treatment reduced DNA double-stranded breaks in motor neurons, highlighting its protective role against ROS-induced damage [12,259,260]. These investigations collectively underscore MitoTEMPO’s potential as a therapeutic treatment for neurodegenerative disorders related to oxidative stress.

Critical appraisal. While these molecules have all proven to be beneficial in cells and animal studies, they have yet to demonstrate any disease modifying effect in humans. There have been no improvements seen with MitoQ after 12 months of administration in PD patients [238], and clinical data for SkQ1 come only from ocular indications. There is currently no data on MitoVitE in models of neurodegeneration. Knowledge of its pharmacokinetics in the brain, ability to reach mitochondria in neuronal cells and show benefit at various doses, and dose tolerability in robust PD, AD ALS or HD models would need to be determined prior to entering into clinical trials. Data for MitoTEMPO in PD, AD and ALS models [257,258,259,260,261] is promising; however, much of the data were collected from toxins or transgenic models and little information is known about how much drug reaches the brain after dosing systemically, and no clinical trials in a neurodegenerative disease have been reported yet.

**Table 3:** Comparison of mitochondria-targeted antioxidants (MTAs) evaluated in neurodegenerative disease models. Supporting references are given for each entry.

MitoTEMPO	MitoVitE	SkQ1	MitoQ	Feature
TPP^+^ + TEMPO nitroxide; SOD mimetic [256,262]	TPP^+^ + vitamin E analogue; tocopherol head group [235,252]	TPP^+^ conjugate; plastoquinone moiety [243,261]	TPP^+^ conjugate; ~500-fold mitochondrial accumulation [230,235]	Targeting Mechanism
Superoxide dismutase-mimetic nitroxide; redox cycling [256,262]	Inhibits lipid peroxidation; protects mitochondrial membrane integrity [252,254,255]	Reduced by the respiratory chain (redox cycling); scavenges O_2_•^−^; inhibits cardiolipin peroxidation [243,261]	Recycles between ubiquinol & ubiquinone; scavenges O_2_•^−^ & ONOO^−^ [235,263]	Primary Antioxidant Mechanism
↓ mitochondrial O_2_•^−^ in Aβ-treated cortical neurons; ↓ tau aggregation [12,259]	No study in AD models identified; protective in cultured cells [253,254]	Partly retards AD-like pathology and cognitive decline in senescence-accelerated OXYS rats [243,264]	↓ Aβ accumulation and lipid peroxidation, and less synaptic loss, in 3xTg-AD mice [240,241]	Evidence in AD Models
↓ H_2_O_2_ and cell death in rotenone-treated neurons; protects dopaminergic neurons in MPTP-treated IDH2-knockout mice [257,258]	Not reported in PD models in the literature reviewed	Not reported in PD models in the literature reviewed	Protects dopaminergic neurons in MPTP and 6-OHDA models [236,237,265]	Evidence in PD Models
↓ DNA double-strand breaks (γH2AX) in motor neurons of Wobbler mice [12,260]	Not reported in ALS models in the literature reviewed	Not reported in ALS models in the literature reviewed	Neuroprotective in SOD1-G93A mice; accumulates in spinal cord and muscle [242]	Evidence in ALS Models
Not reported in HD models in the literature reviewed	Not reported in HD models in the literature reviewed	Not reported in HD models in the literature reviewed	Improved fine motor control and ↓ oxidative damage markers (muscle) in R6/2 mice; protects HD striatal cells [266,267]	Evidence in HD Models
Pre-clinical only; no NDD clinical trials reported [12]	No clinical trials in NDD identified	No NDD trials; phase 2b/3 trials in dry-eye disease only [245,246]	Phase II PD trial (MitoQ-PD): no significant benefit at 12 months [238]	Clinical Trials
Preclinical only; CNS pharmacokinetics not characterised [12]	Data limited to cell models; no in vivo neurodegeneration studies [253,254,255]	Human CNS pharmacokinetics not reported; clinical data limited to ophthalmic use [246]	Human CNS exposure and target engagement not demonstrated; TPP^+^-related toxicity at high doses in mice [12,238,251]	Key Limitations
↓ H_2_O_2_ and cell death in rotenone-treated neurons; protects dopaminergic neurons in MPTP-treated IDH2-knockout mice [257,258]	Small, short, exploratory; adherence; no evidence of disease modification	Pilot randomised trial in PD: improvement in non-motor symptoms [268]	Provides ketone bodies as alternative mitochondrial fuel	Ketogenic diet

↓: Reduced.

### 9.2. Mitochondrial Fusion and Biogenesis Enhancers

SS-31, also known as Elamipretide, Bendavia, or MTP-131, is a mitochondria-targeting tetrapeptide that protects mitochondrial function in AD and PD models by acting as an antioxidant. In dopaminergic neurons, SS-31 reverses mitochondrial dysfunction, which includes decreased oxygen consumption, ATP generation, mitochondrial swelling, and cell death. SS-31 reduces Aβ-induced neurotoxicity by increasing antioxidant enzymes like Prxs and reducing mitochondrial fission protein levels like DRP1 and FIS1. It also increases the expression of mitochondrial biogenesis proteins (PGC-1α, Nrf1/2, and TFAM) and fusion proteins (MFN1 and MFN2) [269]. In APP/PS1 transgenic mice, SS-31 reduces mitochondrial dysfunction by attenuating oxidative stress and correcting the mitochondrial fission/fusion imbalance. Long-term SS-31 treatment reduces β-amyloid plaque formation, reverses synaptic protein loss, and slows the progression of cognitive and behavioral deficits in APP/PS1 transgenic mice [270]. In animal models of oxidative stress-related diseases, BGP-15, a small hydroxylamine compound, has cytoprotective effects by reducing mitochondrial ROS production and inhibiting the JNK/AIF/caspase pathway while activating the AKT/mTOR pathway and extracellular signal-regulated kinases phosphorylation. In particular, BGP-15 promotes mitochondrial fusion by boosting the MFN1, MFN2, and OPA1 levels. It also enhances mitochondrial biogenesis by activating the PGC-1α/Nrf1/TFAM pathway. In AD models, BGP-15 reduces mitochondrial dysfunction, neuronal loss, and dendritic damage while restoring acetylated tau-induced neurotoxicity and cognitive impairments [271].

Critical appraisal. The evidence for fusion and biogenesis enhancers in neurodegeneration comes from cell and rodent models, mostly toxin-induced or transgenic. Mitochondrial readouts such as PGC-1α, MFN and DRP1 levels were measured in tissue, and no study in patients has shown that these changes can be followed or that they track clinical benefit. The regulatory approval of elamipretide is limited to Barth syndrome [272,273] and does not establish efficacy in AD or PD, and no controlled efficacy trial in these diseases was identified in this review.

### 9.3. Mitochondrial Fission Inhibitors

Mitochondrial division inhibitor 1 (Mdivi-1) is a cell-permeable, widely used putative DRP1 inhibitor that decreases DRP1-mediated mitochondrial fission while also providing neuroprotection in AD and PD models. Mdivi-1 inhibits mitochondrial fission, reducing ROS production, MMP loss, and ATP synthesis in Aβ-treated SK-N-SH neuroblastoma cells. Mdivi-1 inhibits Aβ-induced mitochondrial apoptosis by blocking the Cyt c/caspase-9/caspase-3 pathway [274]. Furthermore, Mdivi-1 retains motor function, maintains dopamine levels, and reverses nigrostriatal degeneration in neurotoxicity caused by human A53T α-synuclein overexpression. Overexpression of A53T α-synuclein in rats causes aggregation, toxic phosphorylation, ROS-mediated lipid peroxidation, mitochondrial fragmentation, and dysfunction, all of which are mitigated by Mdivi-1 treatment [275]. However, mdivi-1 was later shown to inhibit mitochondrial complex I reversibly and to modulate ROS production independently of DRP1, so results obtained with this compound alone should be interpreted with caution [14]. P110 is a seven-amino-acid peptide that reduces DRP1 enzymatic activity by preventing its interaction with FIS1 while not interfering with other mitochondrial adaptors. P110 inhibits DRP1 hyperactivation, which reduces mitochondrial fragmentation and damage, lowers ROS generation, and improves MMP and mitochondrial integrity in MPP+-treated SH-SY5Y neuronal cells and primary rat dopaminergic neurons. P110 protects neuronal cells from death in PD models by inhibiting the Bax/Cyt c mitochondrial apoptotic pathway and LC3-mediated autophagy [276]. In MPTP-induced PD mice, P110 protects neurons by inhibiting DRP1-dependent p53 mitochondrial translocation, hence avoiding dopaminergic neurodegeneration. Furthermore, P110 treatment decreases APP and β-site APP-cleaving enzyme 1 expression while enhancing neuroprotective proteins such as disintegrin, metalloproteinase domain-containing protein 10, and klotho in SH-SY5Y neurons. P110 may reduce Aβ generation and improve neuroprotection in AD mice [277,278].

Critical appraisal. Support for fission inhibition is limited to cell and rodent models. The best-known compound, mdivi-1, has DRP1-independent effects on complex I [14], so conclusions drawn from it alone are unreliable, and more selective approaches such as P110 have not been tested in patients. Because DRP1-dependent fission is also needed for normal mitochondrial distribution and quality control, chronic inhibition may carry its own risks, and the therapeutic window in humans is unknown.

### 9.4. Mitochondria-Driven Inflammation Inhibitors

MCC950 is a potent small-molecule inhibitor of NLRP3 inflammasome that targets the ATP-hydrolysis motif within the NACHT domain, thereby inhibiting ATPase activity and impeding NLRP3 inflammasome assembly. Due to its excellent specificity and potency, MCC950 has been widely employed in experimental research on inflammasome-mediated neuroinflammation. MCC950 suppresses α-synuclein-induced inflammasome activation in primary murine microglia, reducing caspase-1, IL-1β, and apoptosis-associated speck-like protein (ASC) release and blocking ASC oligomerisation. MCC950 inhibits caspase-1 activation in mouse models of PD, reducing neurodegenerative processes such as motor impairment, dopaminergic neuronal loss, and α-synuclein aggregation [279,280].

Similarly, OLT1177 (Dapansutrile), an orally accessible β-sulfonyl nitrile molecule, specifically inhibits NLRP3 inflammasome activation. It inhibits NLRP3 ATPase activity, which prevents inflammasome assembly and the activation of caspase-1 and pro-inflammatory cytokines like IL-1β, IL-18, and IL-6. Importantly, OLT1177 may pass the blood–brain barrier and has shown a good safety profile in humans, with current trials focusing on inflammatory disorders including acute gout flares. In APP/PS1 transgenic mouse models of AD, three months of oral OLT1177 treatment restored synaptic plasticity and improved cognitive performance, as indicated by improved learning and memory in the Morris water maze. Treatment decreased Aβ plaque load and altered metabolic indicators, such as deaminated purines and glutathione metabolites. In MPTP-induced PD models, OLT1177 retained motor function, protected against dopaminergic neuronal degeneration, and decreased α-synuclein accumulation and pro-inflammatory cytokine production [281,282,283].

Downstream inhibition of inflammasome signalling has also demonstrated therapeutic potential. VX-765 (Belnacasan) is a selective caspase-1 inhibitor that prevents the release of IL-1β. This orally accessible prodrug is transformed into its active metabolite, VRT-043198, which is non-toxic and can pass the blood–brain barrier. Genetic ablation studies using caspase-1-deficient APP/PS1 mice show that loss of caspase-1 significantly reduces IL-1β activation and microglial inflammation, preserving spatial memory, enhancing amyloid-β clearance, and decreasing amyloid-β deposition, indicating its potential as a neuroprotective anti-inflammatory target [284,285].

In addition to inflammasome inhibition, the cGAS–STING pathway, a key regulator of innate immune responses to cytosolic DNA, is receiving increased attention. TDI-8246 and TDI-6570 are tiny molecules that inhibit human and mouse cyclic GMP-AMP synthase (cGAS), respectively. These compounds have high gastrointestinal absorption and great brain permeability, making them ideal for central nervous system applications. In primary microglia, TDI-6570 inhibits HT-DNA-induced cGAS activation while suppressing cGAS-dependent interferon (IFN) responses in a dose-dependent manner. In tauopathy models, TDI-6570 reduces microglial IFN responses, strengthens neuronal MEF2C transcriptional networks, and protects against synaptic loss and cognitive decline [286].

Further downstream, H-151, a 3-acylaminoindole derivative, functions as a selective and irreversible inhibitor of interferon genes (STING). H-151 covalently binds to STING’s Cys91, blocking palmitoylation and hence reducing type I interferon responses and TBK1 phosphorylation. H-151 therapy in 5xFAD mouse models of AD lowers amyloid-β pathology and neuroinflammation, as demonstrated by the decreased expression of phosphorylated TBK1(p-TBK1), p-p65 and p-IRF3 proteins. Collectively, these pharmacological inhibitors demonstrate the therapeutic potential of targeting inflammasome activation and innate immune signalling pathways to reduce neuroinflammation and impede the course of neurodegenerative illness [287,288]. Innate immune cells like neutrophils have also been shown to be affected by NOS/NO signalling, from their differentiation to oxidative burst and apoptosis [50]. As iNOS and nNOS derived NO has been shown to drive pyroptosis over apoptosis in myeloid cells [52], NOS/NO signalling could also intersect with the inflammasome and cGAS–STING pathways discussed previously, although this has not yet been tested in models of neurodegeneration.

Critical appraisal. Human safety data, where they exist (for example OLT1177), come from inflammatory conditions such as gout, whereas the evidence in neurodegeneration is from mouse models in which inhibitors were often given before or early in pathology. It remains uncertain whether mitochondrial injury is the dominant driver of inflammasome activation in patients, and no pharmacodynamic marker of target engagement in the human brain has been established.

### 9.5. Mitochondrial Permeability Transition Inhibition

Since the discovery of cyclosporin A (CsA) as the first inhibitor of the mitochondrial permeability transition pore (mPTP) over four decades ago, significant evidence has linked mitochondrial malfunction and mPTP opening to apoptosis in neurodegenerative disorders [289]. This has sparked significant interest in developing CsA-related mPTP inhibitors as possible therapeutic medicines. Although many of these inhibitors have shown promising results in preclinical research, their precise molecular processes are yet unknown. This uncertainty stems primarily from the polypharmacological character of CsA and its derivatives, which interact with many molecular targets [290]. Furthermore, research into the specific molecular composition and process of mPTP synthesis remain subjects of ongoing investigation.

Several mitochondrial proteins have been proposed to be involved in mPTP production. Inner mitochondrial membrane (IMM) proteins include the adenine nucleotide translocator (ANT) and the mitochondrial phosphate carrier (PiC), while outer mitochondrial membrane (OMM) proteins include the voltage-dependent anion channel (VDAC) and BCL-2 family proteins (BAK and BAX). However, genetic studies using deletion or overexpression of these proteins have yielded inconsistent results, implying that their roles in pore creation may be indirect or context dependent [291].

Among the proteins investigated, only peptidyl-prolyl cis-trans isomerase, also known as cyclophilin D (CypD), has consistently been identified as a major regulator of mPTP opening. CypD’s peptidyl-proline isomerase (PPIase) activity promotes conformational changes that allow pores to open. Inhibiting CypD reduces cellular sensitivity to Ca^2+^ excess and ROS by inhibiting mPTP production [292]. However, when intracellular Ca^2+^ levels are above a crucial threshold, mPTP opening can occur independently of CypD inhibition. This suggests that CypD operates primarily as a regulatory rather than structural component of the pore. Current theories suggest that mPTP creation begins with the interaction of ANT and CypD within the inner mitochondrial membrane, which, together with other protein components, may stimulate the production of pore-like structures that cross both mitochondrial membranes [291].

The majority of known mPTP inhibitors are CypD-dependent, with cyclosporin A being the most thoroughly researched. CsA therapy protects neurons from oxidative damage in mAPP mouse models of AD that produce mutant human amyloid precursor protein. It reduces ROS formation, corrects Ca^2+^ dysregulation, and prevents mitochondrial swelling. These effects are accompanied by significant enhancements to learning and memory performance. CypD-deficient mAPP animals showed comparable neuroprotective effects, adding to the therapeutic promise of targeting CypD in neurodegenerative disorders [293].

CypD inhibition has also been shown to provide neuroprotection in PD mice. CypD-deficient animals show better cytoprotection than wild-type controls during MPP^+^-induced neurotoxicity experiments. Isolated mitochondria from these animals exhibit increased tolerance to MPP^+^ treatment and lower ROS generation. Furthermore, the expression of apoptotic markers stays constant, indicating that CypD deficiency’s protective effects are most evident under acute mitochondrial stress situations [294].

Despite these promising results, cyclosporin A’s therapeutic applicability as a mPTP inhibitor is hindered by a number of issues, including toxicity, immunosuppressive effects, and low penetration into the central nervous system. As a result, major research efforts are focused on generating safer and more selective CsA replacements. One technique is to use virtual screening and computational drug design to uncover synthetic and semisynthetic compounds with high potential as mPTP inhibitors. These candidates can then be optimised using in silico quantitative structure–activity relationship (QSAR) modelling and molecular docking techniques [295].

Several promising substances have resulted from these experimental efforts. Valasani et al., for example, developed a series of selective CypD inhibitors using diastereomeric crystallisation and pharmacophore modelling [296]. Similarly, Belkacem et al. produced non-peptidic cyclophilin inhibitors with chemical structures different from CsA that inhibit peptidyl-prolyl isomerase [297]. Quinoxaline-derived compounds can inhibit CypD and reduce Ca^2+^-dependent mitochondrial swelling in rat liver mitochondria. These molecules are promising alternatives to CsA, while more research is needed to determine their safety and efficacy in clinical settings [298].

Critical appraisal. Evidence for mPTP inhibition in neurodegeneration comes from the mAPP and MPTP mouse models, and the neuroprotection seen in CypD-deficient animals was most evident under acute mitochondrial stress. The molecular identity of the pore remains unresolved, CsA is limited by toxicity, immunosuppression and poor central nervous system penetration, and the newer CypD inhibitors have not been tested in patients, so their translational potential is unproven.

### 9.6. Mitochondrial Gene Therapy

Significant progress has been achieved in gene therapy for neurodegenerative diseases (NDDs) in recent years, owing to a better understanding of the genetic elements that cause these disorders and breakthroughs in methods for delivering therapeutic DNA or RNA sequences to target tissues [299]. Gene therapy techniques can either mute pathogenic genes, as shown in illnesses caused by gain-of-function mutations, or induce gene overexpression to compensate for loss-of-function mutations. Gene therapy has various advantages over conventional pharmacological treatments, including the ability to target tissues that have previously demonstrated limited responsiveness to traditional therapies [300].

To ensure efficient gene delivery to the central nervous system (CNS), both viral and non-viral vectors have been designed. These vectors can carry transgenes that encode therapeutic proteins, complementary DNA (cDNA) for gene replacement, CRISPR-Cas9/gRNA systems for gene editing, and small interfering RNA (siRNA) for gene silencing [301]. Adeno-associated viruses (AAVs) are the most commonly employed vectors for gene therapy in NDDs because of their low immunogenicity, neuronal tropism, and long-term gene expression. AAVs comprise 13 different serotypes, with AAV2 being one of the most commonly used in clinical research due to its well-established safety profile and effective neural transduction [302]. For example, AAV2-mediated delivery of the nerve growth factor (NGF) gene has been studied as a treatment method for AD via direct injection into the basal forebrain. NGF is a neurotrophic factor that promotes the survival and function of cholinergic neurons, which are especially vulnerable to AD. Despite being well-tolerated in clinical trials, AAV2-NGF therapy did not result in significant changes in cognitive function after 24 months of treatment [303].

Given the crucial role of mitochondrial dysfunction in the pathophysiology of several NDDs, mitochondria-targeted gene therapy has emerged as a possible treatment option. In vitro models of PD and AD have shown neuroprotective effects from gene therapy techniques that regulate mitochondrial ROS generation and mitochondrial dynamics [13]. However, clinical outcomes have been less encouraging, possibly due to the advanced stage of neurodegeneration in patients at the time of treatment initiation. Despite these challenges, current research continues to investigate the therapeutic potential of mitochondria-directed gene treatments, particularly for diseases strongly associated with mitochondrial dysfunction, such as PD and inherited mitochondrial disorders.

Three approaches are relevant to mitochondrial disease and are being explored for neurodegeneration. First, allotopic expression of a mitochondrial gene via AAV has made it to the clinic for Leber hereditary optic neuropathy (LHON), with intravitreal injection of AAV2-ND4 improving vision in both injected and fellow eyes in two phase 3 studies [304]. This would only allow for the treatment of specific mitochondrial genes and is limited by vector size and delivery. Second, mtDNA base editors DdCBE and TALED are able to target specific mutations in mtDNA without creating double strand breaks [305,306]. These have currently only been tested in cell lines and animal embryos and off-target editing must be resolved before clinical use. Third, replacement of mitochondria via pronuclear transfer could avoid passing on mitochondrial disease [307] but does not treat established neurodegeneration. As seen with AAV2-NGF for AD, while the vector was shown to be safe, there was no cognitive gain after 24 months [303], showing that safe delivery does not by itself guarantee clinical benefit when the intervention begins late and does not address mitochondrial dysfunction directly.

### 9.7. Metabolic Modulators

They can help increase the availability of NAD+ or give alternate substrates for damaged mitochondria. Oral nicotinamide riboside has been shown to be safe and increase NAD levels in the brain in PD during the NADPARK phase 1 trial [308]. Whether this will show benefit is yet to be seen, and a placebo-controlled study needs to be performed with clinical outcomes. Another intervention is to give the body another source of energy through ketones. A small, randomised trial in PD showed that patients on a ketogenic diet and those on a low-fat diet had improvements in non-motor symptoms but the ketogenic diet showed more benefit [268]. In both cases, the evidence comes from small, short, exploratory studies, adherence to ketogenic diets is demanding, and there is no evidence yet that these interventions modify disease course.

### 9.8. Safety Considerations

As these drugs will be targeting mitochondria, they will also affect normal mitochondria in non-targeted cells. As TPP+ will build up based on membrane potential, higher doses of lipophilic TPP+ cations will disrupt the electron transport chain and increase proton leak [309], leaving a small therapeutic window as an antioxidant and becoming a pro-oxidant/uncoupler. Commonly used tool compounds can also act on off-targets: Mdivi-1 is commonly used as a DRP1 inhibitor but can act as a reversible complex 1 inhibitor [14]. Clearing all ROS could be detrimental if redox signaling is affected. With gene editing, there could be off-target effects or effects that shift heteroplasmy. Safety assessment should therefore include mitochondrial function in non-target tissues, long-term dosing, and for chronic indications, tolerability data.

## 10. Critical Synthesis and Recent Advances

### 10.1. Cause, Consequence or Amplifier? Grading the Evidence

Mitochondrial abnormalities are detectable in all four diseases, but the evidence that they initiate, rather than accompany, neurodegeneration differs markedly. We distinguish three tiers of evidence: (i) human genetic causation, in which mutation of a mitochondrial or mitochondria-associated gene is sufficient to cause disease; (ii) human observational evidence, in which mitochondrial defects are present in patient tissue or biofluids, and ideally, precede overt neuronal loss; and (iii) model-based evidence, in which mitochondrial defects have been demonstrated only in cells or animals. In PD, tier (i) evidence is strong: loss-of-function mutations in PINK1 and PRKN cause autosomal recessive parkinsonism [150,151], mtDNA deletions accumulate in nigral neurons with age and reach higher levels in PD [153,154], and respiratory-chain deficiency restricted to dopamine neurons is sufficient to produce progressive parkinsonism in mice [157]. In ALS and frontotemporal dementia, mutations in CHCHD10 link disease directly to mitochondrial cristae organisation and respiratory function [159], and loss of C9orf72 destabilises complex I assembly [161]. In AD, in contrast, the evidence is mostly tiers (ii) and (iii): reduced mtDNA content and oxidative damage are found in patient brains [135,139], and low cerebrospinal fluid mtDNA has been reported in preclinical AD [145], but no mitochondrial gene causes typical late-onset AD and the mitochondrial cascade hypothesis remains a hypothesis [139]. In HD, mutant huntingtin is the primary insult, and some structural neuronal changes appear to precede measurable respiratory defects (Section 8), which suggests that mitochondrial impairment may amplify, rather than trigger, striatal degeneration. Table 4 summarises the evidence of mitochondrial involvement by disease specific evidence tier.

Two recent findings caution against over-simplified models. First, basal mitophagy in vivo proceeds largely independently of PINK1 in mouse tissues with high metabolic demand [15] and is only minimally affected by loss of Pink1 or parkin in Drosophila [16]. These observations suggest that the PINK1–Parkin pathway operates mainly as a stress-response pathway and that its deficiency does not equate to a global failure of mitochondrial turnover, which weakens the assumption that every mitophagy defect is a shared therapeutic target. Second, the widely used pharmacological DRP1 inhibitor mdivi-1 also acts as a reversible inhibitor of mitochondrial complex I and modulates ROS production [14], so part of the neuroprotection attributed to DRP1 inhibition in earlier studies (Section 9.3) may reflect off-target effects on respiration and should be interpreted with caution.

### 10.2. Selective Neuronal Vulnerability

A central unresolved question is why ubiquitous organelles cause the loss of anatomically restricted neuronal populations. Proposed determinants include the high bioenergetic and Ca^2+^ load imposed by autonomous pacemaking and extensive axonal arborisation in SNpc dopaminergic neurons [186], the metabolic demand and mitochondrial Ca^2+^ sensitivity of striatal MSNs (Section 8), and the extreme axonal length of motor neurons, which magnifies the demand on mitochondrial transport and local ATP supply [113,114]. These explanations are plausible but remain largely correlative, because most supporting data derive from rodent tissue and cultured neurons. Studies that apply single-nucleus and spatial methods to human post-mortem brains are needed to test whether vulnerable neurons differ in mtDNA burden, respiratory-chain composition or mitochondrial Ca^2+^ handling before degeneration begins.

Retinal ganglion cells (RGCs) illustrate the same principle outside the brain. The intraretinal axon is very long and mostly non-myelinated, meaning that they have a large metabolic cost. Heterozygous mutations in OPA1, which reduce inner membrane fusion (see Section 4.2), lead to dominant optic atrophy and the degeneration of RGCs even though OPA1 is expressed in all tissues [311]. Mutations in SLC25A46 also cause a similar optic atrophy phenotype [110]. Familial glaucomatous neurodegeneration provides a complementary genetic example: whole exome sequencing of 9 families with MYOC negative primary open angle glaucoma or primary angle closure glaucoma yielded some novel candidate genes that are enriched in RGCs [312]. These candidates were found to be more enriched in extracellular matrix pathways than mitochondrial ones. Hence, the study shows how genetic variants may converge on a vulnerable neuronal population but does not implicate mitochondria by itself; whether mitochondrial dysfunction contributes to RGC loss in familial glaucoma remains to be tested.

These hypotheses can be tested with emerging technologies. Single nucleus RNA-Seq of human SN has allowed us to identify a dopaminergic population that is selectively lost in PD [313]. Using spatial transcriptomics and proteomics, we can map mitochondrial gene programs onto anatomy, and the single-cell sequencing of mtDNA has been used to track lineages [314]; this could allow us to look at the levels of heteroplasmy and deletions within cells that can be identified. If applied to patients at an early stage or even pre-symptomatic, these techniques could help determine whether a population of cells is at higher risk due to increased mitochondrial burden before degeneration.

### 10.3. Recent Advances (2023–2026)

Mitochondria as drivers of neuroinflammation. Cytosolic mtDNA is a danger signal linked to NLRP3 inflammasome activation [94], and loss of PINK1 or Parkin exacerbates STING-dependent inflammation in mice [158], providing a mechanistic link between defective mitophagy and neuroinflammation in PD. In tauopathy and amyloid models, microglial cGAS–STING activation contributes to synaptic and cognitive deficits [286,288], and inflammasome signalling is increasingly viewed as a convergence point of neurodegenerative pathology [95]. Glial mitochondria are therefore emerging as therapeutic targets in their own right [315], which widens the scope of mitochondria-directed therapy beyond neurons.

Mitophagy as a druggable node. Genetic loss or pharmacological inhibition of the mitochondrial deubiquitinase USP30, which opposes PINK1–Parkin-dependent ubiquitylation, increased mitophagy, reduced phosphorylated α-synuclein and attenuated nigral dopaminergic neuron loss in an α-synuclein mouse model [310]. The brain-penetrant USP30 inhibitor MTX325 entered a phase 1 first-in-human trial in healthy volunteers and PD patients in 2024 [316]. In AD models, stimulating mitophagy or mitochondrial proteostasis reduced amyloid-β and tau pathology and improved memory [141,143]. Because basal mitophagy can be PINK1-independent [15], patient selection and pharmacodynamic readouts of mitophagy will be critical for interpreting these trials.

Intercellular mitochondrial transfer. Microglia can exchange organelles, including mitochondria, with neurons through tunnelling nanotubes. In co-culture, microglia transferred aggregated α-synuclein and tau out of burdened neurons, and donated healthy mitochondria that reduced oxidative stress and restored neuronal function; nanotube-mediated transfer of aggregates was compromised in microglia carrying the LRRK2 G2019S or TREM2 T66M and R47H variants [317]. These findings connect mitochondrial quality control to glial genetics and provide a rationale for the transfer strategies discussed in Section 11.3, although selective delivery of intact organelles to vulnerable neuronal populations remains unsolved.

First regulatory approval of a mitochondria-targeted peptide. In September 2025, the U.S. Food and Drug Administration granted accelerated approval to the cardiolipin-binding tetrapeptide elamipretide (SS-31) for Barth syndrome, a primary mitochondrial disease [272]; the supporting clinical data are a randomised crossover trial in 12 participants followed by an open-label extension of up to 168 weeks [273]. This is a regulatory precedent for mitochondria-targeted drugs, but because the approval rests on a muscle-strength endpoint in a rare mitochondrial cardioskeletal disorder, it is not evidence of efficacy in neurodegeneration (Section 9.2).

Mitochondrial transplantation. Not only can mitochondria be exchanged between microglia and neurons, but astrocytes have also been shown to donate working mitochondria to neurons, which can aid their survival following focal cerebral ischaemia in mice [318]. This has provided evidence that mitochondria can be transferred between cells in the CNS. Translation to chronic neurodegeneration faces unresolved problems: the source, viability and dose of isolated mitochondria, delivery to vulnerable neurons across the blood–brain barrier, and immune responses to allogeneic mitochondria and their mtDNA.

Using patient-derived induced pluripotent stem cell (iPSCs) to study mitochondria allows for the study of human cells with known genotypes. iPSC derived neurons with the A53T mutation in α synuclein were shown to have increased nitrosative stress associated with reduced MEF2–PGC-1α signalling and mitochondrial dysfunction [48]. Such models complement animal studies; however, they primarily represent immature neurons and lack key features of ageing, including age-related changes in glial cells and neural circuit organisation. These limitations restrict their ability to fully model late-onset neurodegenerative diseases.

### 10.4. Why Have Mitochondria-Targeted Therapies Failed Clinically?

Despite strong preclinical data, no mitochondria-directed therapy has yet shown disease-modifying benefit in a large randomised trial in AD, PD, ALS or HD. High-dose coenzyme Q10 showed no benefit in early PD [319] or in HD [184], creatine monohydrate was stopped for futility in PD [320], and MitoQ did not slow PD progression over 12 months [238]. Several non-exclusive explanations emerge from this review: (i) intervention occurs too late, after substantial neuronal loss; (ii) CNS exposure and target engagement are rarely demonstrated because most trials lacked a mitochondrial pharmacodynamic biomarker; (iii) mitochondrial defects are probably dominant in only a subset of patients, for example, carriers of PINK1/PRKN variants or those with a high somatic mtDNA burden, whereas trials enrol unselected populations; (iv) tool compounds may act off-target, as illustrated by mdivi-1 [14]; and (v) in some diseases, mitochondrial dysfunction may be a downstream amplifier, so that correcting it cannot halt an upstream proteinopathic driver. Encouragingly, oral nicotinamide riboside was safe and increased cerebral NAD levels in a phase 1 trial in PD [308], illustrating that target engagement can now be measured in the human brain even though efficacy remains untested. Table 5 summarises the clinical status of the main strategies discussed in this review.

Two further barriers deserve emphasis: blood–brain barrier penetration and trial design. As many of these TPP^+^ conjugates have been designed for use in the periphery, little data exist on their ability to penetrate the brain once administered systemically. Neurodegenerative disease is a heterogeneous group of diseases, therefore, enrolling patients with no selection criteria could mask an effect in patients who may have a mitochondrial driven disease. If there is no biomarker demonstrating that a drug is exerting its intended pharmacodynamic effect, it can be difficult to determine whether treatment failure is due to the drug being inactive or to inadequate target engagement. We believe that treating patients at the prodromal or pre-symptomatic stage, using biomarkers or genotyping to identify appropriate patients, together with evidence that the drug has reached and engaged its target, will increase the likelihood of treatment success.

## 11. Future Directions

Future directions can be divided into four categories: (i) the use of biomarkers to identify and stratify patients; (ii) identifying mechanisms of selective vulnerability; (iii) delivery/transfer and gene editing methods; and (iv) treatments and clinical trials.

### 11.1. Candidate Mitochondrial, Autophagy and Mitophagy Biomarkers for Diagnosis, Prognosis and Stratification

Establishment of candidate mitochondrial biomarkers in blood and cerebrospinal fluid, including cell-free mtDNA, mitochondrial damage-associated molecular patterns, extracellular vesicle mitochondrial content, and bioenergetic indices enabling presymptomatic identification and disease surveillance in neurodegenerative disorders. Integration with existing biomarker panels, such as amyloid/tau in AD and neurofilament light, merits consideration. For example, reduced cerebrospinal fluid mtDNA has been reported in preclinical AD [145], and extracellular vesicles carry mitochondrial cargo in AD [144].

Markers in blood and CSF that are linked to mitochondrial stress, mitophagy and autophagy could aid in the diagnosis, prognosis and stratification of patients. However, none have been validated for use in the clinic. Cell free mtDNA has received the most attention. Reduced levels of CSF mtDNA have been found in preclinical AD [145] and PD patients in the early stages of disease [321]. This may be due to loss of mitochondrial mass prior to cell loss. However, as it is found in CSF, this limits its use in clinical practice, and we do not know whether it is specific, as mtDNA in the extracellular environment can also reflect inflammation and cell death. It has also been shown that CSF mtDNA is lowered in the late stages of progressive MS [322] but increased in MS patients and normalized after treatment with autologous haematopoietic stem cells [323].

As extracellular vesicles (EV)s will protect their content and contain mitochondrial components including mitochondrial DAMPs in AD [144], measuring mitochondrial/mitophagy-related proteins in blood-derived EVs is appealing. However, current methods of isolation and enriching for neuronal-derived EVs have not been established, and these markers are still being explored. Measuring mitochondrial function in peripheral blood cells is available, but how this relates to the brain is unknown. Neurofilament light chain is known to increase in HD [183] as well as other neurodegenerative diseases and should be used as the comparison for any mitochondrial markers. Before clinical use, each marker needs standardized pre-analytical handling and assays, reference ranges that account for age and sex, and prospective validation of sensitivity, specificity, and prognostic value in independent cohorts (Table 6).

Markers of autophagy and mitophagy are less developed. LC3 and p62/SQSTM1 are standard tissue readouts of autophagy, but a single static measurement does not distinguish increased autophagic flux from blocked clearance [324]. Lysosomal proteins measured in neuron-derived plasma extracellular vesicles were altered in preclinical AD [325], and phospho-Ser65-ubiquitin, a product of PINK1 activity, can be measured by ELISA in human cells, autopsy brain and blood samples [326], but diagnostic or prognostic value has not been shown for these markers. The circulating mitochondrial-stress markers FGF21 and GDF-15 are elevated in primary mitochondrial disease [327,328]; they are not specific, can also rise with ageing and in other conditions, and have not been validated in AD, PD, ALS or HD. Priorities are paired CSF and plasma measurements, longitudinal sampling to test prognostic value, and testing whether a marker can identify patients likely to respond to mitophagy-directed therapy.

**Table 6:** CSF mitochondrial biomarkers: Evidence, limitations and readiness.

Readiness	Main Limitations	Evidence	Biomarker
Exploratory	Requires CSF for most findings; direction depends on disease and stage; non-specific; assay variability	Reduced in preclinical AD [145], early PD [321] and end-stage progressive MS [322]; increased in MS and normalised after transplantation [323]	Cell-free mtDNA (CSF, plasma)
Exploratory	Isolation not standardised; cell of origin uncertain; no outcome data	Mitochondrial DAMPs in EVs in age-related disease and AD [94,144]	Mitochondrial DAMPs and EV mitochondrial cargo
Exploratory	Genotype-restricted; not tested in sporadic disease	Reported in PRKN/PINK1 parkinsonism [158]	Mitochondrial damage-associated inflammatory markers
Most advanced; use as comparator	Marks axonal injury; not mitochondria-specific	Rises with disease burden in HD and predicts progression [183]	Neurofilament light chain (comparator)
Exploratory	Isolation of neuronal EVs not standardised; not specific to mitochondria	Altered lysosomal proteins in plasma neuron-derived EVs in preclinical AD [325]	Autophagy–lysosome proteins in neuron-derived EVs
Exploratory	Diagnostic or prognostic value not established; reflects PINK1 activity	Detectable by ELISA in cells, autopsy brain and blood [326]	Phospho-Ser65-ubiquitin (mitophagy marker)
Exploratory in neurodegeneration	Not validated in neurodegeneration; non-specific; influenced by age and other conditions	Raised in primary mitochondrial disease [327,328]	FGF21 and GDF-15 (circulating mitochondrial-stress markers)

### 11.2. Mitochondrial Susceptibilities of Cellular Phenotypes

Future study must elucidate the reasons behind the selective vulnerability of neuronal populations (e.g., SNpc dopaminergic neurons in PD, striatal MSNs in HD, motor neurons in ALS, hippocampal/neocortical neurons in AD) to mitochondrial dysfunction. Progress in single-nucleus RNA sequencing, spatial transcriptomics, and mitochondrial proteomics of post-mortem brain tissue will be essential.

### 11.3. Mitochondrial Transplantation and Transfer Therapies

Direct mitochondrial transfer by tunnelling nanotubes, extracellular vesicles, or microinjection constitutes novel methodologies for reinstating mitochondrial function in deteriorating neurons. Preclinical proof-of-concept studies suggest additional investigation in neurodegenerative disease models. Microglia-to-neuron organelle exchange through tunnelling nanotubes has been described [317].

### 11.4. Mitochondrial-Targeted Therapies and the Blood–Brain Barrier

The unsuccessful outcomes of promising candidates, such as MitoQ in clinical trials for PD, highlight the urgent necessity for improved central nervous system delivery methods. Key research areas encompass nanoparticle-based delivery systems, targeted ultrasound-mediated blood–brain barrier (BBB) opening, and conjugation with brain-penetrant vectors. Mitochondria-targeted nanotherapeutics for neurodegenerative disease are under active development [329].

### 11.5. Mitochondrial Genome Editing

The emergence of CRISPR-independent mitochondrial base editors, such as DdCBE and TALED [305,306], capable of editing heteroplasmic mtDNA mutations, paves the way for novel therapeutic strategies for inherited mitochondrial illnesses and somatic mtDNA mutations that accumulate in neurodegenerative diseases.

### 11.6. Therapeutic Strategies Addressing Multiple Targets

Considering the intricacy and redundancy of mitochondrial dysfunction in neurodegenerative diseases, combinatorial therapeutic strategies that concurrently target oxidative stress, mitophagy, calcium dysregulation, and neuroinflammation may be more efficacious than single-target therapies. Combination medications should be systematically developed via systems pharmacology modeling.

Some concrete combinations could be made from these mechanisms. A mitophagy enhancing drug such as a USP30 inhibitor [310,316] and an NAD^+^ precursor [308] could be used in patients with impaired mitophagy or PINK1/PRKN variants. Another example would be a mitochondria targeted antioxidant or NLRP3 inhibitor in combination with a disease modifying therapy for proteinopathies in early AD. This would allow for the treatment of mitochondrial and inflammatory pathways while targeting the primary pathology. Trial design should include a factorial or adaptive platform design to separate the contribution of each component, a pharmacodynamic readout of mitochondrial target engagement for each agent, enrichment by genotype or biomarker, early disease stage, and prespecified monitoring for overlapping toxicities, particularly for TPP^+^-based compounds.

### 11.7. Artificial Intelligence in Mitochondrial Pharmacotherapy

A rapidly developing subject is the application of machine learning algorithms to identify novel mPTP inhibitors, mitophagy modulators, and mitochondrial-targeted drug delivery systems. The incorporation of cryo-EM structural data, transcriptome datasets, and clinical trial outcome data inside AI frameworks may facilitate the rapid discovery of viable therapeutic targets.

### 11.8. Sex, Age and Ancestry

Mitochondria are known to vary in males and females as well as age and ethnicity. These characteristics are typically left out when looking at mitochondrial-based therapies. There are many studies that have shown mitochondria in the brain to differ between males and females. These include but are not limited to respiration, oxidative stress, Ca^2+^ handling, and injury responses. It has been suggested that estrogen may signal to the mitochondria and cause some of these changes. More studies need to be conducted to see whether these changes can help explain susceptibility to disease and responses to treatments [330]. As people age, their mtDNA accumulates more somatic deletions, and this is higher in PD patients [153,154]. Mitochondrial quality control is also affected by age [126]. People that inherit different forms of mtDNA have been linked to lower risks of PD [331]. There are certain mtDNA haplogroup clusters that have been shown to protect against PD in individuals of European ancestry. More preclinical studies should be conducted with both males and females. When studying humans, we should also take note of their gender, age, and race/haplogroup and see whether there is a difference in treatment.

## 12. Conclusions

Mitochondria are crucial to the coordination of neuronal energy metabolism, calcium signaling, redox homeostasis, and cell death regulation. This review summarises evidence that mitochondrial dysfunction, including impaired oxidative phosphorylation, disrupted fission–fusion dynamics, defective mitophagy, abnormal calcium buffering and increased oxidative damage, is a common pathological mechanism in AD, PD, ALS and HD, despite their distinct genetic etiologies and neuroanatomical specificity.

Disease-specific examinations reveal that although the final phenotype of neuronal death is the same, the initial triggers and the prevailing mitochondrial mechanisms are dramatically different. Amyloid beta accumulation, tau hyperphosphorylation and reduced mitophagy interact with each other to form a self-perpetuating cycle in which mitochondrial dysfunction acts as an early amplifier in AD. Mutations in the PINK1–Parkin pathway in PD demonstrate the critical dependence of dopaminergic neurons on efficient mitochondrial quality control. The pathogenesis of ALS is characterized by an imbalance of fission and fusion as well as the disturbed respiratory chain assembly, caused by mutations in SOD1, FUS, and C9orf72, which exacerbates motor neuron degeneration. HD: striatal medium spiny neurons are selectively vulnerable because they depend preferentially on oxidative phosphorylation and are sensitive to mitochondrial malfunction triggered by mutant huntingtin. In HD, mutant huntingtin is the primary insult, and mitochondrial dysfunction acts as an amplifier.

Grading the evidence shows that a causal mitochondrial role is best supported in PD and in genetic forms of ALS and frontotemporal dementia, whereas in sporadic AD and HD, mitochondrial impairment is better viewed as an early amplifier of a proteinopathic process (Section 10.1). This distinction matters for translation: mitochondria-targeted interventions are most likely to succeed in genetically or biomarker-defined subgroups, and are less likely to succeed when applied to unselected patients late in the disease course (Section 10.4).

The therapeutic landscape has expanded, but no mitochondria-targeted therapy has yet shown disease-modifying benefit in a large randomised trial in AD, PD, ALS or HD. Preclinical research has shown promise for mitochondria-targeted antioxidants, fission inhibitors, fusion and biogenesis enhancers, mPTP inhibitors and inflammasome modulators. Nevertheless, the translation gap remains substantial: clinical trial failures, notably with MitoQ in PD, underscore the challenges of brain bioavailability, patient stratification, intervention timing, and heterogeneity of disease stage. These issues require more sophisticated trial designs that incorporate mitochondrial biomarkers, genotype-based patient stratification and earlier therapeutic interventions.

In summary, mitochondria are not passive victims of neurodegenerative disease but active contributors whose causal weight varies from primary driver in PD and genetic ALS/FTD to early amplifier in sporadic AD and HD. Improvements in single-cell omics, structural biology, targeted drug delivery, and better mechanistic understanding of mitochondrial biology offer promising avenues for the development of next-generation disease-modifying therapies for neurodegenerative illnesses.

## Figures and Tables

**Table 1:** Comparison of the main regulated cell-death pathways: triggers, mitochondrial and redox/nitrosative links, and evidence in neurons and glia.

Evidence in Neurons and Glia	Mitochondrial and Redox/Nitrosative Link	Triggers and Executioners	Pathway
Trophic-factor withdrawal in neurons [78]; transient NMDA/NOS activation in cortical neurons [92]	MOMP is the commitment step; NOS activation can trigger permeability transition and delayed apoptosis [92]	Ca^2+^ overload, DNA damage, ROS, trophic-factor withdrawal; BAX/BAK, cytochrome c, caspase-9/-3 [71,72,78]	Apoptosis
Necroptosis effectors activated in AD brain and AD models [96]	Mitochondrial ROS enhance RIPK1 autophosphorylation [81]	TNFα, RIPK1/RIPK3/MLKL; caspase-8 inhibition [80,81]	Necroptosis
Ferroptotic mechanisms in dopaminergic cell models of PD [97]	Mitochondrial ROS and redox imbalance [82]	Iron overload, lipid peroxidation, GPX4 loss [82]	Ferroptosis
NLRP3 activation in microglia in AD models [93]	mtDNA and mitochondrial ROS activate NLRP3 [86]; NOS isoform and NO output shift apoptosis versus pyroptosis in K562 cells [52]	DAMPs/PAMPs; NLRP3–caspase-1; gasdermin D/E [83,84,85,91]	Pyroptosis
PAR drives α-synuclein neurodegeneration in PD models [98]	Ca^2+^ and ROS/RNS-induced DNA damage; AIF released from mitochondria [87]	PARP1 overactivation, PAR polymer, AIF [87,88]	Parthanatos

**Table 2:** Comparative mitochondrial dysfunction across the four major neurodegenerative diseases.

Huntington’s Disease (HD)	Amyotrophic Lateral Sclerosis (ALS)	Parkinson’s Disease (PD)	Alzheimer’s Disease (AD)	Feature
HTT (≥36 repeats; reduced penetrance commonly occurs at 36–39 repeats) [172]	SOD1, TDP-43, FUS, C9orf72 [9,166]	PINK1, PRKN, LRRK2, SNCA [150,151]	APP, PSEN1, PSEN2, APOE ε4 [133,134]	Primary Genetic Factors
Striatal medium spiny neurons [172]	Upper & lower motor neurons [185]	Dopaminergic SNpc neurons [186]	Hippocampal & cortical neurons [133]	Affected Cell Type
Complex II & IV impairment [174,187]	Complex I & IV dysfunction [9,161,166]	Complex I inhibition [11,188]	Complex IV deficiency [139,189]	OXPHOS Defect
↑ from mHTT- impaired ETC & NRF2 suppression [174,190]	↑ from mutant SOD1 & impaired antioxidant defence [160,163]	↑ via complex I inhibition & α-synuclein [8,11]	↑ from Aβ & tau interaction with mitochondria [138,139]	ROS Production
mHTT impairs PGC-1α & TFEB; reduced mitophagy [127,174,191]	TDP-43/FUS disrupt fission- fusion dynamics [192,193]	PINK1/Parkin mutations; α-syn blocks mitophagy [150,156]	Aβ & tau block PINK1/Parkin; impaired TFEB [142,143,194]	Mitophagy Defect
mHTT sensitises IP_3_R; mitochondrial Ca^2+^ overload [179,180,195]	EAAT2 loss → excitotoxic Ca^2+^ overload [166,196]	α-syn disrupts ER–mitochondria contacts and Ca^2+^ homeostasis [197,198]	ER-mitochondria Ca^2+^ transfer disrupted by PS1 [102,199]	Ca^2+^ Dysregulation
Mitochondrial genome instability via oxidative stress [178,200]	Oxidative DNA damage in affected tissue [163,201]	mtDNA deletions in SNpc; somatic mutations ↑ with age [153,154]	↑ 8-OHdG; reduced mtDNA copy number [135,202]	mtDNA Damage
mHTT disrupts DRP1 GTPase; excessive fission [175,203]	DRP1/FIS1 ↑; MFN1/2 & OPA1 ↓; excessive fission [165,166,204]	↑ DRP1 activation by α-syn; impaired MFN1/2 [104,105,205]	↑ DRP1 fission; ↓ MFN2 fusion; fragmented networks [206,207]	Fission/ Fusion Balance
R6/2, YAC128, zQ175 knock-in mouse models [208,209,210]	SOD1-G93A, TDP-43 & C9orf72 mice [211,212,213]	MPTP, rotenone, PINK1 & Parkin knockout mice [147,148,214,215]	3xTg-AD, 5xFAD, APP/PS1 mouse models [216,217,218]	Key Animal Models
↓ striatal glucose metabolism (PET); mHTT in CSF [176,219,220]	↑ CSF neurofilament light; impaired mitochondrial respiration in blood cells [221,222]	↓ striatal F-DOPA uptake; α-syn seed amplification positive in CSF (total CSF α-syn ↓); ↓ platelet complex I activity [223,224,225,226]	↓ CMRglc (PET); ↓ CSF Aβ42 and ↑ tau; ↓ CSF mtDNA copy number [145,227,228]	Candidate/Research Biomarkers

↑: Increased, ↓: Decreased.

**Table 4:** Representative evidence for mitochondrial involvement by disease, model or sample, and evidence tier.

Evidence Tier and Reference	Model or Sample	Key Finding	Disease
Tier ii [135,137]	Post-mortem human brain	Reduced neuronal mtDNA copy number, increased heteroplasmy and early oxidative damage to mitochondrial protein and DNA	AD
Tier iii [138,139,141,142,143]	Mice, worms and cultured cells	Aβ and tau impair mitophagy, mitochondrial proteostasis and ETC activity; restoring mitophagy improves pathology	AD
Tier ii–iii [132]	APP/PSEN1 mice and human AD brain	Mitochondrial plaques: clusters of mitochondria in dystrophic neurites with lysosomal dysfunction	AD
Tier ii [145]	Human CSF (biomarker study)	Lower CSF mtDNA in preclinical disease	AD
Tier i [150,151]	Human genetics	Loss-of-function PINK1 and PRKN variants cause autosomal recessive parkinsonism	PD
Tier ii [153,154]	Human tissue	Somatic mtDNA deletions accumulate with age and are higher in PD	PD
Tier iii [310]	α-Synuclein mouse model	USP30 inhibition increased mitophagy and reduced nigral neuron loss	PD
Clinical, negative [238]	Randomised clinical trial	MitoQ gave no benefit after 12 months	PD
Tier iii [169]	Cell and in vivo models	Preventing TDP-43 localisation to mitochondria reduced toxicity	ALS/FTD
Tier ii–iii [170]	Patient iPSC-derived motor neurons	Poly(GR) from C9orf72 causes mitochondrial dysfunction, oxidative stress and DNA damage	ALS/FTD
Tier iii [165]	Transgenic ALS mouse	Increased FIS1 and DRP1 with decreased MFN1 and OPA1 from early symptomatic stages	ALS
Clinical [171]	Phase 3 trial	Tofersen lowered SOD1 and neurofilament but not a clinical outcome at 28 weeks (not mitochondria-directed)	ALS
Tier ii [176]	Presymptomatic patients (PET, 1H-NMR)	Reduced striatal glucose uptake and pyruvate utilisation before motor symptoms	HD
Tier iii [177,178]	HD mouse models	MSN dendritic alterations precede respiratory defects	HD
Tier ii–iii [182]	HD models and human samples	Mutant huntingtin binds and activates DRP1	HD
Clinical, negative [184]	2CARE clinical trial	High-dose coenzyme Q10 did not slow functional decline	HD

**Table 5:** Clinical status and principal limitations of mitochondria-targeted strategies discussed in this review.

Principal Limitation	Best Available Human Evidence	Mechanistic Rationale	Strategy/Agent
Brain target engagement not demonstrated; no biomarker-based patient selection	Phase 2 in early PD (n = 128, 12 months): no slowing of progression [238]	Scavenges mitochondrial ROS; recycled by the respiratory chain	MitoQ (TPP^+^-conjugated ubiquinone)
Unselected patients; no mitochondrial pharmacodynamic readout	No benefit in early PD (QE3) [319] or HD (2CARE) [184]	Electron carrier and antioxidant	Coenzyme Q10
Not specific to mitochondrial defects	PD trial stopped for futility [320]	Supports cellular energy buffering	Creatine
Efficacy untested	Phase 1 in PD: safe; increased cerebral NAD levels [308]	Restores NAD^+^ for OXPHOS, sirtuin signalling and mitophagy	Nicotinamide riboside (NAD^+^ precursor)
Approval concerns a primary mitochondrial disease, not neurodegeneration	Accelerated approval in Barth syndrome [272]; no efficacy data in AD or PD	Binds cardiolipin; stabilises cristae and respiratory supercomplexes	Elamipretide (SS-31)
Efficacy unknown; basal mitophagy may be PINK1-independent [15]	Phase 1 in healthy volunteers and PD [316]; protection in an α-synuclein mouse model [310]	Enhances PINK1–Parkin-dependent mitophagy	USP30 inhibition (MTX325)
Mdivi-1 inhibits complex I [14]	Preclinical only	Limits excessive fission	DRP1 inhibition (mdivi-1, P110)
Immunosuppression and poor CNS penetration (Section 9.5)	Preclinical only [293]	Prevents permeability transition and Ca^2+^-induced swelling	mPTP/CypD inhibition (cyclosporin A)
Neuronal delivery; off-target editing	Preclinical only [305,306]	Corrects pathogenic mtDNA variants	mtDNA base editing (DdCBE, TALED)
Neuronal delivery; off-target editing	Preclinical only [305,306]	Extracellular vesicle	EV
Neuronal delivery; off-target editing	Preclinical only [305,306]	Mitochondria-associated ER membrane	MAM
Neuronal delivery; off-target editing	Preclinical only [305,306]	Multiple sclerosis	MS
Neuronal delivery; off-target editing	Preclinical only [305,306]	Neurofilament light chain	NfL
Neuronal delivery; off-target editing	Preclinical only [305,306]	Nitric oxide	NO
Neuronal delivery; off-target editing	Preclinical only [305,306]	Nitric oxide synthase (nNOS, neuronal; iNOS, inducible; eNOS, endothelial)	NOS
Neuronal delivery; off-target editing	Preclinical only [305,306]	Retinal ganglion cell	RGC
Neuronal delivery; off-target editing	Preclinical only [305,306]	Reactive nitrogen species	RNS

## Data Availability

No new data were created or analyzed in this study. Data sharing is not applicable to this article.

## Abbreviations

The following abbreviations are used in this manuscript

AD	Alzheimer’s disease
ALS	Amyotrophic lateral sclerosis
APP	Amyloid precursor protein
ATP	Adenosine triphosphate
DRP1	Dynamin-related protein 1
ETC	Electron transport chain
HD	Huntington’s disease
IMM	Inner mitochondrial membrane
MCU	Mitochondrial calcium uniporter
mHTT	Mutant huntingtin protein
MPTP	1-methyl-4-phenyl-1,2,3,6-tetrahydropyridine
mPTP	Mitochondrial permeability transition pore
MTAs	Mitochondria-targeted antioxidants
mtDNA	Mitochondrial DNA
NLRP3	NOD-, LRR- and pyrin domain-containing protein 3
OMM	Outer mitochondrial membrane
OXPHOS	Oxidative phosphorylation
PD	Parkinson’s disease
PINK1	PTEN-induced kinase 1
ROS	Reactive oxygen species
